# Deep learning for sub-ångström-resolution imaging in uncorrected scanning transmission electron microscopy

**DOI:** 10.1093/nsr/nwaf235

**Published:** 2025-06-05

**Authors:** Zanlin Qiu, Yuan Meng, Junxian Li, Yanhui Hong, Ning Li, Xiaocang Han, Yu Liang, Wing Ni Cheng, Guolin Ke, Linfeng Zhang, Weinan E, Xiaoxu Zhao, Jin Zhang

**Affiliations:** School of Materials Science and Engineering, Peking University, Beijing 100871, China; School of Materials Science and Engineering, Peking University, Beijing 100871, China; School of Materials Science and Engineering, Peking University, Beijing 100871, China; DP Technology, Beijing 100080, China; School of Materials Science and Engineering, Peking University, Beijing 100871, China; School of Materials Science and Engineering, Peking University, Beijing 100871, China; School of Materials Science and Engineering, Peking University, Beijing 100871, China; School of Materials Science and Engineering, Peking University, Beijing 100871, China; DP Technology, Beijing 100080, China; DP Technology, Beijing 100080, China; AI for Science Institute, Beijing 100084, China; AI for Science Institute, Beijing 100084, China; Center for Machine Learning Research, Peking University, Beijing 100871, China; School of Mathematical Sciences, Peking University, Beijing 100871, China; School of Materials Science and Engineering, Peking University, Beijing 100871, China; AI for Science Institute, Beijing 100084, China; School of Materials Science and Engineering, Peking University, Beijing 100871, China; Center for Nanochemistry, Beijing Science and Engineering Center for Nanocarbons, Beijing National Laboratory for Molecular Sciences, College of Chemistry and Molecular Engineering, Peking University, Beijing 100871, China; School of Advanced Materials, Peking University Shenzhen Graduate School, Shenzhen 518055, China

**Keywords:** transmission electron microscopy, artificial intelligence, super-resolution imaging, denoising diffusion probabilistic model

## Abstract

Achieving sub-ångström resolution has long been restricted to sophisticated aberration-corrected scanning transmission electron microscopy (AC-STEM). Recent advances in computational super-resolution techniques, such as deconvolution and electron ptychography, have enabled uncorrected STEM to achieve sub-ångström resolution without the need for delicate aberration correctors. However, these methods have strict requirements for sample thickness and thus have yet to be widely implemented. In this study, we introduce SARDiffuse—a deep-learning diffusion model designed to enhance spatial resolution and correct the noise level of uncorrected STEM images. Trained with experimental AC-STEM data, SARDiffuse has the capability to restore high-frequency information of STEM images, enabling sub-ångström resolution in an uncorrected microscope. We demonstrate the effectiveness of the model on representative materials, including silicon, strontium titanate and gallium nitride, achieving substantial improvements (<1 Å) in spatial resolution. Detailed statistical analysis confirms that SARDiffuse reliably preserves atomic positions, demonstrating that it is a powerful tool for high-precision material characterization. Furthermore, SARDiffuse effectively mitigates spherical-aberration-induced artifacts, outperforming current methods in artifact correction. Meanwhile, the background information of images, such as thickness variation or carbon contamination distribution, is also preserved. This work highlights the potential of deep learning to realize sub-ångström-resolution imaging in the uncorrected electron microscope, offering a cost-effective alternative to delicate AC-STEM when imaging conventional single crystals.

## Teaser

This study presents a deep-learning diffusion model that processes uncorrected scanning transmission electron microscopy images, achieving atomic resolution in standard crystals without complex hardware, enabling precise and cost-effective atomic-scale material analysis.

## INTRODUCTION

Over the past few decades, aberration-corrected (scanning) transmission electron microscopy (AC-(S)TEM) has become an essential tool for unveiling the nanoworld in materials research, facilitating the study of the atomic structure [1–4], nanoscale composition [3–5], local bonding, defect topology [6–9] and electromagnetic structures [7,8,10–12] of nanomaterials, pushing down the research dimension into the sub-ångström regime. Compared with conventional TEM, the primary advantage of AC-STEM is the ability to provide atomic-resolution Z-contrast imaging. The sub-ångström electron beam, focused by advanced aberration correctors, makes it well suited for atomic-resolution structural and compositional characterization [12,13]. Despite providing sub-ångström spatial resolution, the use of aberration correctors increases the cost, complexity and operational intricacies of AC-STEM, limiting its accessibility for widespread sub-ångström characterization [14].

Before the invention of aberration correctors, many attempts, such as increasing the accelerating voltage of the electron gun and developing computation-based methods, were made to realize sub-ångström spatial resolution with an uncorrected STEM [14–20]. Increasing the accelerating voltage reduces the wavelength of the electron beam, thereby improving the spatial resolution of the STEM [21]. Sub-ångström resolution can be achieved on a 1250-kV STEM even for thicker samples [15]. However, ultra-high voltage requires complex and expensive high-voltage units, significantly increasing the investment and maintenance costs. Moreover, ultra-high-energy e-beams unavoidably introduce knock-on damage when rastering the samples [16]. Because of these drawbacks, ultra-high-voltage STEM has become less popular and modern commercial STEM chooses an intermediate voltage (60–300 kV) [16]. Under this accelerating voltage, the primary limitation to spatial resolution is the spherical aberration (C_3_) of the magnetic lens [16,21].

Computational methods, including maximum entropy deconvolution [17–20], Richardson–Lucy deconvolution [19] and electron ptychography reconstruction [14], have enabled sub-ångström spatial resolution in uncorrected STEM, offering low-cost alternatives for sub-ångström imaging [14,17–20]. The principle of deconvolution methods is that the annular-dark-field STEM (ADF-STEM) signal is approximately proportional to the convolution of the point spread function (PSF) with the object function and the PSF is strongly related to the spherical aberration C_3_ [17–20]. Deconvolution methods can exclude the impact of C_3_ and obtain a ‘most likely’ object function, thereby achieving sub-ångström resolution [17–20]. However, deconvolution methods are not robust, as they may fail to eliminate artifacts resulting from spherical aberration [17–20]. Meanwhile, shot noise, sample tilt and other possible factors might influence the deconvolution results [17]. Additionally, the deconvolution principle assumes that the multiple scattering effects in STEM imaging are negligible [17–20], which becomes problematic for thick samples, for which such effects cannot be ignored.

Besides, electron ptychography methods have achieved sub-ångström spatial-resolution imaging with an uncorrected STEM [14] using iterative reconstruction methods based on 4D-STEM datasets to eliminate the effect of aberrations, particularly C_3_ [14,22]. The integration of a multislice method to address the multiple scattering effects into the electron ptychography reconstruction algorithm enables the reconstruction of relatively thick bulk materials, thereby unveiling the 3D crystal structure [23,24]. Currently, multislice ptychography methods have achieved an in-plane spatial resolution of 10–20 pm, enabling the imaging of complex crystal structures and defects inside the crystals [23–25]. Recent results show that a spatial resolution of 0.5 Å has been successfully achieved in an uncorrected STEM by using electron ptychography methods [14]. However, unlike ADF-STEM, electron ptychography has not been widely adopted due to its complexity, time-consuming nature and strict requirements for sample thickness [25]. Therefore, there is a clear need to develop relatively straightforward and universally applicable sub-ångström imaging methods designed for uncorrected STEM.

Deep-learning (DL) methods have demonstrated significant potential for addressing the super-resolution tasks of electron microscopy owing to their data-driven nature [26,27]. Over the past decade, many DL methods, involving convolutional neural networks [27], deep residual attention networks [28], generative adversarial networks [29] and diffusion networks [30,31], have been adopted to increase the resolution of scanning electron microscopy (SEM) images and STEM images. Resolution improvement of two to four times has been successfully attained [27,29,30]. In addition, unlike electron ptychography that requires 4D-STEM datasets, DL methods can work well with the widely accessible ADF-STEM images [27], showing their potential for universal sub-ångström-resolution imaging for uncorrected STEM. However, most previous works have focused on DL super-resolution methods for SEM images and achieved super-resolution through up-sampling the images [28–30] (e.g. transforming the image dimension from 256 × 256 to 512 × 512). The up-sampling methodology is not suitable for uncorrected atomic-resolution STEM images, as their spatial resolution is always several times larger than their pixel lengths. Besides, this methodology requires paired training data with ground-truth labels, which are not available for uncorrected STEM images, as acquiring sub-ångström-resolution STEM images by using an uncorrected STEM is challenging. To date, there is one DL super-resolution model designed for atomic-resolution STEM—AtomSegmentNet [27]. This model uses synthetic STEM images, generated by STEM simulations with background noise, to train the U-Net models [27]. The ground truths are the noise-free simulated STEM images with deep sub-ångström resolution (<50 pm). Although the trained models can greatly improve the spatial resolution of aberration-corrected ADF-STEM images, the model performance on uncorrected STEM images has not been tested [27]. To sum up, DL-based methods show great potential for sub-ångström-resolution imaging and have achieved widespread adoption, but existing DL models might not be suitable for processing uncorrected ADF-STEM images. Therefore, there is an urgent need to develop specific super-resolution DL models for the real-time transformation of uncorrected STEM to sub-ångström-resolution STEM images.

Herein, we propose Sub-Ångström-Resolution-Diffuse (SARDiffuse)—a denoising diffusion probabilistic model (DDPM)-based framework, designed to increase the spatial resolution of STEM images acquired from an uncorrected STEM to the sub-ångström regime. Diffusion models have demonstrated exceptional performance in numerous computer vision domains such as super-resolution [30,32–35], image generation [35–38], image inpainting [35,37] and pattern recognition [39]. In addition, recent studies further suggest that diffusion models exhibit distinct advantages in denoising and carrying out super-resolution tasks for conventional electron microscopy images [30]. In this work, we trained SARDiffuse by using low-rank adaptation (LoRA) fine-tuning methods (see Supplementary materials for more details) with experimental atomic-resolution AC-STEM images as training data. After training, SARDiffuse successfully enhances the spatial resolution (information-transfer limit) of the uncorrected ADF-STEM images captured from conventional uncorrected STEM machines (∼136 pm) to better than 100 pm, which is the hallmark for aberration-corrected STEM. The underlying mechanism of improving spatial resolution is attributed to the inherent capability of the diffusion model to restore high-frequency information. Statistical analysis demonstrates that the processed STEM images greatly resemble the uncorrected STEM images, with atomic-position deviations confined to 1.87 ± 0.74 pixels, corresponding to 17.77 ± 7.03 pm. The background information as well as the drifting information are well preserved. More importantly, SARDiffuse effectively corrects the spherical-aberration-induced artifacts presented in the uncorrected STEM images. The codes are available at https://github.com/dptech-corp/SARDiffuse.

## RESULTS AND DISCUSSION

**Figure 1.**
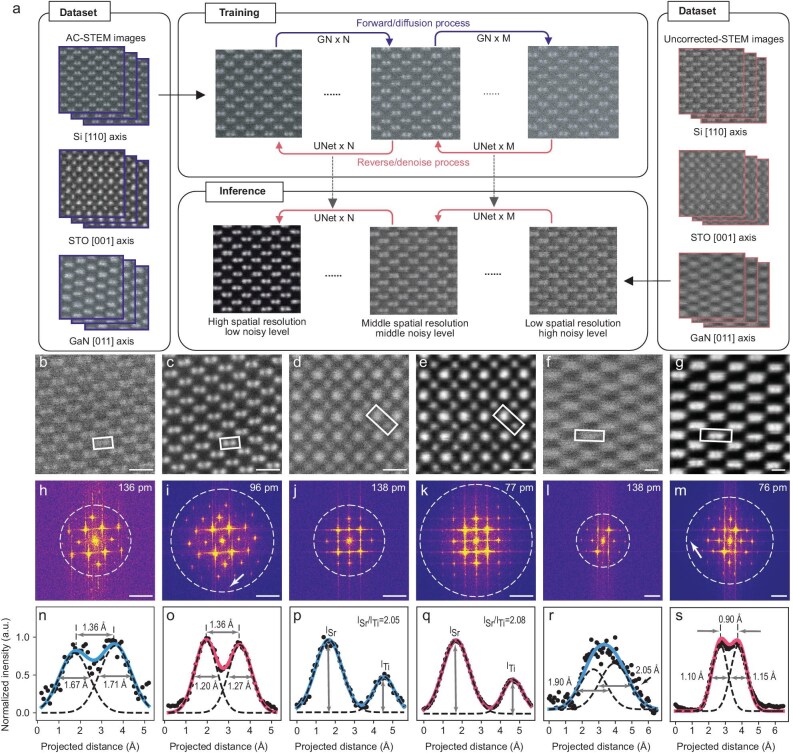
Sub-ångström-resolution ADF-STEM images generated by SARDiffuse from uncorrected STEM images. (a) Schematic of SARDiffuse. During the training stage, experimental AC-STEM images with sub-ångström resolution are used as training data. In the forward-diffusion process, Gaussian noise is added to the AC-STEM images to generate noisy data, while the reverse-diffusion process utilizes a DL network (U-Net: Fig. S2) to restore the original AC-STEM images. In the inference stage, SARDiffuse processes the raw ADF-STEM images acquired by using the uncorrected microscope to achieve sub-ångström resolution. (b, c) Raw and processed ADF-STEM images of silicon (Si) [110]. (d, e) Raw and processed ADF-STEM images of strontium titanate (STO) [100]. (f, g) Raw and processed ADF-STEM images of gallium nitride (GaN) [101]. (h–m) FFT patterns of (b–g), respectively. The information-transfer limit is marked by the white dotted circles. The white arrows in (i) and (m) also mark the information-transfer limit. (n–s) Intensity line profiles extracted from the white rectangles in (b–g), respectively. Gaussian fits (colored lines and black dashed lines in (n–q)) were applied to Si [110], STO [100] and GaN [101] intensity line profiles to indicate the full width of the atom at half maximum (FWHM) and the atomic contrast. The FWHM of raw and processed Si [110] images are measured to be 1.67 and 1.20 Å, respectively. The relative contrast (*I*_Sr_/*I*_Ti_) of raw and processed STO [001] images are measured to be 2.05 and 2.08, respectively. For GaN [101], Ga dumbbells can be resolved in the processed image while they appear as an indistinct ellipse cloud in the raw image. Scale bars: (b–e) 5 Å; (f, g) 2 Å; (h–m) 5 nm^-1^.

The SARDiffuse framework consists of three major stages: data acquisition, model training and inference stages (Fig. 1a). During the data-acquisition stage, AC-STEM images are first acquired in an aberration-corrected electron microscope. Subsequently, these images are used to fine-tune the pretrained DDPM models (see Supplementary materials for more details). The training of the SARDiffuse model involves two processes: forward diffusion and backward diffusion. Forward diffusion iteratively adds the Gaussian noise to the AC-STEM images with random steps of between 0 and 1000, causing the images to degrade into noisy images. The backward diffusion process trains the model (Fig. S2) to recover the original images from the noisy images by utilizing prior knowledge of the noise-addition process and the Bayesian theorem [37] (see Methods and Supplementary materials for more details). After training, SARDiffuse is ready to perform super-resolution tasks of uncorrected STEM images through iterative inference. In the inference stage, when given an input STEM image acquired at an uncorrected STEM and the inference steps, SARDiffuse can generate plausible images with improved spatial resolution.

To demonstrate the performance, validity and robustness of our SARDiffuse, we used experimental AC-STEM images (Fig. S3) of silicon (Si) [110], strontium titanate (STO) [100] and gallium nitride (GaN) [101] as training datasets to train the model. Si [110], having a dumbbell-like structure with a Si dumbbell distance of 136 pm [40], was frequently used to justify the spatial resolution of the electron microscope. In addition, STO, revealing a signature perovskite ABO_3_ structure, was selected because it served as an ideal sample to evaluate the performance of our model in distinguishing contrast patterns in STEM images. As shown in previous literature, the contrast of STEM images is significant, as it has a power-law relationship with the atomic number Z (∼Z^1.6–2^) [41–43]. Analogous to Si [110], GaN [101] exhibits a dumbbell-like structure, but the distance between the Ga dumbbells is significantly smaller (∼91 pm). Therefore, we utilized three representative samples to validate the super-resolution capability of SARDiffuse.

After completing the training of SARDiffuse models, ADF-STEM images of Si [110] (Fig. 1b), STO [100] (Fig. 1d) and Ga [101] (Fig. 1f) were acquired by using an uncorrected electron microscope (JEOL-F200, see Methods). These three images (Fig. 1b, d and f) were subsequently processed by SARDiffuse and we can notice a significantly improved spatial resolution or information-transfer limit (Fig. 1c, e and g) after inference, as suggested by the fast Fourier transformation (FFT) power spectrum (Fig. 1h–m) and the intensity line profile (Fig. 1n–s and Fig. S4). For clarity, we refer to the images acquired at an uncorrected STEM (Fig. 1b, d and f) as raw images and images after model processing (Fig. 1c, e and g) as processed images. A comparison between raw and processed ADF-STEM images of Si [110] (Fig. 1b and c) reveals the full width at half maximum (FWHM) of Si atomic columns decreases from ∼167 to ∼120 pm (Fig. 1n and o, and Fig. S5), confirming the improved spatial resolution. However, FWHM is not an ideal metric for evaluating spatial resolution, as it is an indirect metric and is significantly affected by the noise in ADF-STEM images (Fig. 1n and o, and Fig. S5). Direct assessment of spatial resolution by using ADF-STEM images of Si [110] may be challenging, as the distance between silicon dumbbells is fixed at 136 pm [40]. The information-transfer limit is a good alternative for evaluating the spatial resolution [25]. The information-transfer limits of raw and processed ADF-STEM images of Si [110] are calculated to be 136 and 96 pm according to the FFT patterns of raw and processed images (Fig. 1h and i), undoubtedly supporting the improvement of spatial resolution.

Using the same approach, the sub-ångström resolution of STEM imaging was also achieved in STO [100] and GaN [101] (Fig. 1d and g). Notably, the information-transfer limits of raw and processed ADF-STEM images of STO [100] (Fig. 1d and e) were determined to be 138 and 77 pm, respectively, as validated by the FFT patterns (Fig. 1j and k). As atomic numbers have a power-law relationship with the contrast of atomic columns, it is crucial that any super-resolution methods do not alter the relative contrast of each atomic column [41–43]. The intensity-line-profile evaluation of STO atomic columns was conducted before and after processing (Fig. 1p and q). Sr and Ti atomic columns were used to evaluate the contrast difference between raw and processed images, as oxygen atomic columns are usually invisible in ADF-STEM images due to its small scattering cross section [44,45]. The relative contrasts of Sr and Ti atomic columns (*I*_Sr_/*I*_Ti_) are ∼2.05 and ∼2.08 for raw and processed images (Fig. 1p and q, and Fig. S5), demonstrating that SARDiffuse does not induce any contrast variations. As for GaN [101], the information-transfer limits of raw and processed ADF-STEM images (Fig. 1f and g) are 138 and 76 pm (Fig. 1l and m). Ga dumbbells can be partially resolved in processed images (Fig. 1g), whereas they appear as an indistinct ellipse cloud in the raw images (Fig. 1f), as confirmed by the intensity line profiles (Fig. 1r and s), suggesting that SARDiffuse increases the spatial resolution of uncorrected ADF-STEM images of Ga [101] to ∼90 pm, i.e. the Ga dumbbell distance. Therefore, our model can both achieve sub-ångström spatial resolution and improve the information-transfer limit—important criteria justifying the resolution performance of SARDiffuse models.

In addition to spatial-resolution improvement, SARDiffuse significantly enhances the signal-to-noise (S/N) ratio, as suggested in the raw and processed images (Fig. 1b–g) as well as the FFT patterns (Fig. 1h–m). The improved S/N ratio is attributed to the denoising nature of the DDPM model [37]. In theory, SARDiffuse can both increase the spatial resolution or information-transfer limit of images and denoise the images, making it optimal for processing uncorrected STEM images. It may be curious that SARDiffuse exhibits excellent super-resolution capability, but this is an unsupervised model and high-resolution ground truth is not available. To explore the underlying mechanisms of super-resolution, the forward and backward diffusion processes are investigated. During the iterative forward-diffusion process, the Gaussian noise addition gradually destroys the high-frequency information, leading to a reduction in the information-transfer limits (Fig. S6). In the reverse-diffusion process, in order to restore the original AC-STEM images, the DL network learns to recover the high-frequency information of STEM images by utilizing prior knowledge of the noise-addition process and the Bayesian theorem [34,37,38] (see Methods). Recovering high-frequency information of STEM images is fairly similar to improving the information-transfer limits or the spatial resolution. Therefore, the training of SARDiffuse involves teaching the DL networks to recover the high-frequency information and thus achieve spatial-resolution improvement, explaining the extraordinary super-resolution capability of SARDiffuse.

**Figure 2.**
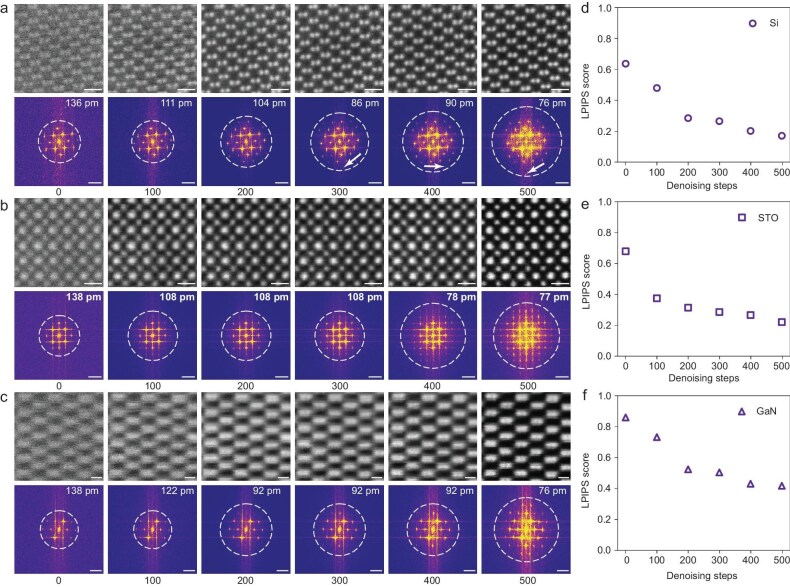
Performance of SARDiffuse as a function of inference steps. (a–c) Processed ADF-STEM images and corresponding FFT power spectrums of (a) silicon [110], (b) STO [001] and (c) GaN [101] under 0, 100, 200, 300, 400 and 500 inference steps, respectively. The information-transfer limit is marked by the white dotted circles. (d–f) LPIPS similarity evaluation for the uncorrected (0 inference step) and the processed ADF-STEM images of (d) Si [110], (e) STO [001] and (f) GaN [101], respectively, as a function of the inference steps. Scale bars: 5 Å for all real space images and 5 nm^-1^ for FFT images.

The super-resolution ability of SARDiffuse can be precisely controlled by the inference steps (Fig. 1a). Notably, introducing additional inference steps improves the information-transfer limits and reduces the noise levels according to the processed images and the corresponding FFT pattern (Fig. 2a–c and Fig. S7). For ADF-STEM images of silicon [110], 300 inference steps are required to reach sub-ångström spatial resolution (Fig. 2a). For ADF-STEM images of STO [100] (Fig. 2b) and GaN [101] (Fig. 2c), the required inference steps are 400 and 200 steps, respectively. In all three cases, 500 inference steps yield an information-transfer limit of ∼76 pm, which is almost identical to the spatial resolution of our training data (experimental AC-STEM images: Fig. S3). Increasing inference steps to >500 steps can further improve the spatial resolution, but the processed image might be untrustworthy, as some atoms in the images are diminished (Fig. S7). This might be attributed to the unsupervised characteristic of SARDiffuse. Therefore, the number of inference steps needs to be carefully evaluated or compromised based on specific types of images to balance the spatial resolution and fidelity of the images.

To evaluate the effect of the inference steps, quantitative comparisons were conducted via the Learned Perceptual Image Patch Similarity (LPIPS) metric—a human perceptual similarity judgment—to compare the raw and processed images with experimental AC-STEM images (training images, Fig. S3) [46]. Although experimental AC-STEM images are not the corresponding ground truths of the raw or processed images, they are the best available alternatives. The LPIPS scores (Fig. 2d) for Si [110] ADF-STEM images gradually decrease from ∼0.63 (raw images) to ∼0.17 (processed images) after 500 inference steps, suggesting that additional inference steps produce processed images that are more analogous to the AC-STEM. A similar trend of LPIPS score versus inference steps has also been observed for STO [100] (Fig. 2e) and GaN [101] (Fig. 2f) based on corresponding ADF-STEM images. The LPIPS score for STO [100] gradually decreases from 0.67 to 0.22 after 500 inference steps (Fig. 2e), whereas the LPIPS score for GaN [101] gradually decreases from 0.85 to 0.41 (Fig. 2f). All these results strongly support that SARDiffuse is indeed consecutively transforming the uncorrected STEM images into AC-STEM images.

**Figure 3.**
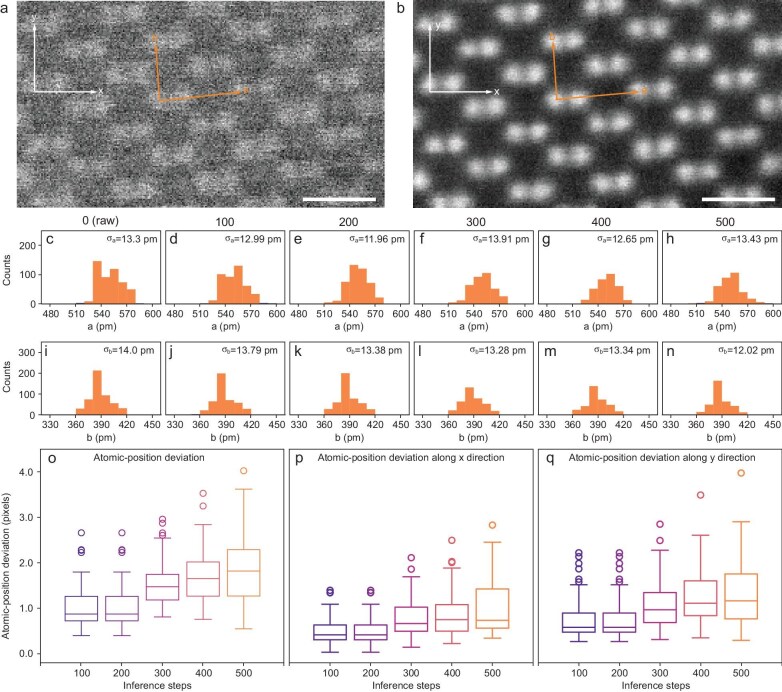
Validity and robustness of SARDiffuse. (a) Raw and (b) processed ADF-STEM images of Si [110]. The inference step is selected as 350 steps. The orange arrows in (a) and (b) mark the two lattice vectors (denoted as a and b lattices) of Si[110] and the white arrows in (a) and (b) mark the x and y directions. (c–n) Histogram of measured lattice lengths, involving (c–h) lengths of the a lattice vector and (i–n) lengths of the b lattice vector of a processed ADF-STEM image after 0, 100, 200, 300, 400 and 500 inference steps. The inference steps are labeled on top of the histograms. The standard deviation of the measured lengths is the precision of the processed images and is labeled at the top-right corner of the corresponding histograms. (o) Box plot of atomic-position deviation as a function of the inference steps. The atomic-position deviation is calculated by generating 50 plausible images for a given uncorrected STEM image and a given inference step. The atomic-position deviation is calculated based on the atomic positions between these 50 plausible images and the original uncorrected STEM image. (p, q) Box plot of atomic-position deviation along the (p) x direction and (q) y direction, respectively, as a function of the inference steps. Scale bars: 5 Å.

Another important aspect of validity is whether SARDiffuse will change the relative location (or the atomic positions). To justify this important criterion, precision examinations and the atomic-position comparison are first conducted by using uncorrected (raw) and processed ADF-STEM images of Si [110] (Fig. S8). The precision can be calculated by measuring the column-to-column spacing, i.e. the a and b lattice parameters (Fig. 3a) [27]. The procedure involves first determining the atomic-position coordinates (x, y directions are labeled in Fig. 3b) by using localized maximum and 2D Gaussian fitting methods [47]; then measuring the column-to-column spacing on uncorrected (Fig. 3c and i) and processed images with inference steps of 100 (Fig. 3d and j), 200 (Fig. 3e and k), 300 (Fig. 3f and l) 400 (Fig. 3g and m) as well as 500 (Fig. 3h and n); and finally calculating the standard deviation of the measurements (see Methods for more details). Both precisions along the a and b directions are ∼12–14 pm, regardless of the inference steps. Considering that the raw images have a pixel length of 9.5 pm, 12–14 pm of precision suggests that the precision is within one or two pixels, which is reasonable, as the scanning noise might easily generate errors of several pixels. Therefore, we can conclude that our precision results validate that SARDiffuse will not change the true precision of the images.

It is noteworthy that SARDiffuse can generate more plausible outputs, as DDPM is a probability model [37] (see Methods for more details). A robust super-resolution model must ensure that the atomic positions of the raw and processed images are preserved, with minimal deviation across all plausible solutions. Therefore, the robustness of SARDiffuse should be carefully examined. To further validate the robustness and precision of our model, 50 plausible processed images are generated for 100, 200, 300, 400 and 500 inference steps as the input. The atomic-position information of both the raw input and all generated images is obtained by using localized maximum and 2D Gaussian fitting methods, and then the deviation of the atomic positions is calculated (see Methods for more details) [47]. The box plot (Fig. 3o) clearly shows that a higher deviation in atomic positions corresponds to more inference steps. The deviation, calculated and presented in Table S1, increases from 1.01 ± 0.45 pixels (9.5 ± 4.3 pm) after 100 steps to 1.87 ± 0.74 pixels (17.8 ± 7.0 pm) after 500 steps. This increase in deviation can be explained, as additional inference steps might result in extra uncertainties or errors. The average deviation along the X direction (Fig. 3p) is all smaller than the average deviation along the Y direction (Fig. 3q). This might be ascribed to the fact that X is the fast-scanning direction and Y is the slow-scanning direction during the acquisition of STEM images. To sum up, the results of the atomic-position deviation highlight that the SARDiffuse model is a robust super-resolution model.

The robustness testing also establishes a criterion for determining the maximum number of inference steps. Users can tailor the precision or accuracy according to their requirements by defining a threshold for the permissible deviation (see Supplementary materials for more details). This criterion provides a clear benchmark for balancing the inference steps and result accuracy. In the meantime, the information-transfer limit can still be utilized to determine the minimum inference steps. Typically, sub-ångström resolution (<100 pm) is desired and there would be a minimum number of inference steps to achieve sub-ångström resolution (Fig. 2 and Fig. S7). Within the range of minimum and maximum inference steps, the spatial resolution and the deviation of the atomic positions should be compromised. Therefore, it is challenging to propose an optimized inference step, as it is highly dependent on the image quality and/or noise level.

**Figure 4.**
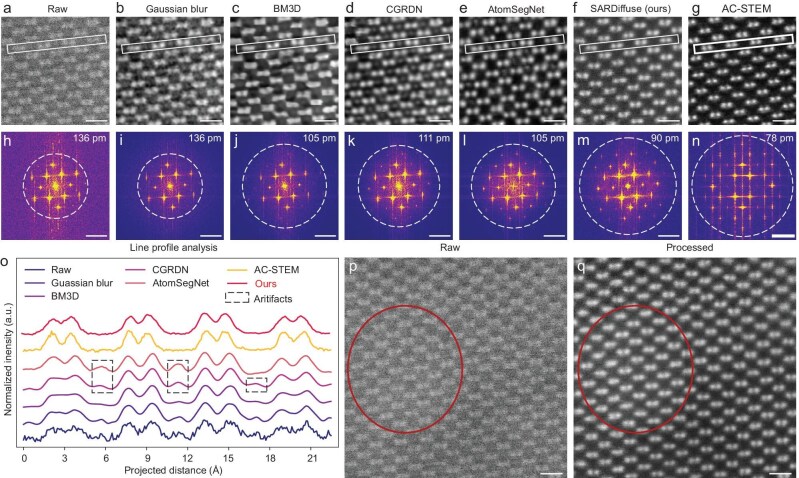
Performance of SARDiffuse and other existing denoising or super-resolution models. (a) Uncorrected ADF-STEM image of Si [110]. (b–f) Processed ADF-STEM image using (b) Gaussian blur, (c) BM3D [48], (d) CGRDN [49], (e) AtomSegNet [27] and (f) SARDiffuse. (g) Aberration-corrected ADF-STEM image of Si [110]. (h–n) Corresponding FFT patterns of (a–g), respectively. The information-transfer limit is marked by the dotted circles. (o) Line intensity profile extracted from the white rectangular box in (a–g). Artifacts can be visualized in (a–e) as indicated by the dashed boxes in (o). Our SARDiffuse methods can avoid the formation of artifacts and generate processed images that are analogous to aberration-corrected ADF-STEM images. (p) Raw and (q) SARDiffuse-processed ADF-STEM images. The circles mark the region with the higher lowest (background) signal. Scale bars: (a–g, p, q) 5 Å; (h–n) 5 nm^-1^.

A comparison with existing denoising or super-resolution methods (including both non-DL-based and DL-based methods) was conducted to evaluate the performance of our SARDiffuse models. An uncorrected ADF-STEM image of Si [110] (Fig. 4a) was selected as the raw input and the AC-STEM images of Si[110] (Fig. 4g) were selected as standard images for comparison. Denoising or super-resolution methods, such as Gaussian blur, BM3D [48], CGRDN [49], AtomSegNet [27] and our SARDiffuse methods, were employed to process the raw input (Fig. 4a). The ADF-STEM images processed by all of the above five methods (Fig. 4b–f) show a lower noise level than the input image (Fig. 4a), confirmed by FFT patterns (Fig. 4h–m). Regarding spatial resolution (or the information-transfer limit), the Gaussian blur method maintains 136 pm (Fig. 4i), while BM3D, CGRDN, AtomSegNet and SARDiffuse achieve spatial resolutions of 105 (Fig. 4j), 111 (Fig. 4k), 105 (Fig. 4l) and 90 pm (Fig. 4m), respectively. Notably, AtomSegNet offers multiple modes such as denoise, denoise plus background subtracted, circular mask segmentation and super-resolution modes [27]. The performance of all these four modes on the same raw input has been tested (Fig. S9). The circular mask segmentation mode (Fig. S9d) and the super-resolution mode (Fig. S9e) can increase the spatial resolution to 70 (Fig. S9j) and 40 pm (Fig. S9k), respectively.

Although AtomSegNet seems to have a better super-resolution capability than our method, artifacts between the silicon dumbbells can be observed in ADF-STEM images processed by AtomSegNet (Fig. 4e and o, and Fig. S9b–e). These artifacts are present in the uncorrected ADF-STEM images (Fig. 4a and o) while they cannot be observed in AC-STEM images of Si [110] (Fig. 4g). Previous literature has reported that the artifacts between the Si dumbbells can be observed by using uncorrected STEM under some imaging conditions (e.g. under specific defocus) [17,18]. Therefore, this artifact is highly subject to spherical aberration [18]. To demonstrate the presence of these artifacts between the Si dumbbells (marked by the black dashed box in Fig. 4o) in uncorrected ADF-STEM images, a multislice STEM simulation was also conducted. When C_3_ is ∼0.5 mm, artifacts between the Si dumbbells emerge in the simulated ADF-STEM images of Si [110] (Fig. S10b). These artifacts would result in incorrect structure analysis and therefore should be fully eliminated [18]. From this aspect, BM3D (Fig. 4c), CGRDN (Fig. 4d) and AtomSegNet (Fig. 4e and Fig. S9b–e) are still problematic for uncorrected STEM images, as they fail to correct the artifacts between the Si dumbbells. Our SARDiffuse methods generate atomic-resolution STEM images that contain no artifacts between the Si dumbbells (Fig. 4f and Fig. S9). This might be attributed to the fact that SARDiffuse is trained by experimental AC-STEM images (Fig. S3) and thus the model recognizes the pattern of experimental AC-STEM images. To sum up, SARDiffuse is currently the only method capable of simultaneously enhancing spatial resolution and removing artifacts when transforming uncorrected STEM images into sub-ångström STEM images.

For the sake of the training efficiency and requirements of pretrained DDPM models, SARDiffuse is trained by 256 × 256 pixel patches of AC-STEM images (see Methods for more details). This requires that the input of the inference stage should also have dimensions of 256 × 256 pixels. However, STEM images usually have dimensions of 512 × 512, 1024 × 1024 or 2048 × 2048 pixels. To address this issue, we crop STEM images into 256 × 256 pixel patches that are processed individually by SARDiffuse and subsequently stitched together. A weighted-averaging stitching algorithm (see Supplementary materials for more details) is introduced to avoid the formation of edge-induced artifacts (stitched lines, see Fig. S11a), simultaneously preserving the accurate atomic position and key sample information, including background and drift details (Fig. S11). As suggested by the uncorrected ADF-STEM images (Fig. 4p), the background intensity is typically inhomogeneous across all of the images due to carbon contamination or thickness variations [50]. Regardless of the cause of contrast variations, the background intensity of the ADF-STEM image reveals sample characteristics and thus any image-processing methods should preserve the background information. As shown in the processed ADF-STEM images (Fig. 4q and Fig. S12), the weighted-averaging methods show their potential to preserve the background information of the original raw image. The background information of ADF-STEM images can be extracted by applying strong Gaussian blurring (standard deviation: 20) to filter out the atomic columns (Fig. S12c and d). The background of the processed image (Fig. S12c) is analogous to the background of the raw input (Fig. S12d). Meanwhile, the sample drift observed in the original uncorrected ADF-STEM image is maintained after processing by SARDiffuse and weighted-averaging stitching (Fig. S11b and c), indicating that the atomic-position information is effectively preserved by our method.

To sum up, SARDiffuse demonstrates its potential to replace costly aberration correctors and bypass complex aberration-correction procedures. While recent advancements in automated aberration-correction algorithms have significantly simplified the correction process [21,51], the reliance on expensive aberration correctors to achieve sub-ångström resolution persists, thereby hindering the progress of materials research. The success of SARDiffuse provides a cost-effective pathway to achieve sub-ångström resolution, facilitating materials research and broadening the scope of experimental possibilities. Further refinements to SARDiffuse are expected to enhance its generalizability, unlocking its potential for sub-ångström-resolution characterization of materials with complex crystal structures and defect configurations. Currently, the model relies on specific types of AC-STEM images for training and fine-tuning—a limitation that could be addressed by expanding the diversity of the training datasets or incorporating simulation-based methods for training data generation. In addition to C_3_ aberration, astigmatism (A_1_) and other anisotropic aberrations significantly affect the spatial resolution of uncorrected STEM images. However, correcting these aberrations is sophisticated due to their anisotropic nature. Future integration of advanced stable diffusion models into SARDiffuse could improve its generative capabilities and enable the correction of different orders of astigmatism in uncorrected STEM images. Continued development of SARDiffuse will thus pave the way for widespread sub-ångström-resolution STEM imaging.

## CONCLUSION

In summary, we have proposed SARDiffuse—a DL model that enables sub-ångström imaging in uncorrected STEM images. Our results demonstrate that SARDiffuse significantly increases the spatial resolution of uncorrected STEM images while simultaneously preserving atomic-position information. Furthermore, SARDiffuse effectively mitigates artifacts induced by spherical aberration, outperforming existing denoising and super-resolution techniques. Notably, SARDiffuse is advantageous because it operates with less expensive uncorrected microscopes. These advantages have the potential to significantly accelerate the widespread adoption of sub-ångström-resolution characterization, making high-precision imaging more accessible across various research and industrial applications. Ongoing development aims to refine the model, enabling the characterization of materials with complex crystal structures and defect configurations, and to correct a broader range of aberrations, significantly expanding the applications of uncorrected electron microscopy to the nanoworld.

## METHODS

### STEM characterization

AC-STEM images were acquired by using an aberration-corrected JEM-ARM200F (NEOARM) equipped with a cold field emission gun operating at 200 kV and a fifth-order ASCOR aberration corrector. The convergence semiangle of the electron probe was ∼30 mrad. The collection angles for ADF-STEM imaging were between 62 and 280 mrad. ADF-STEM images of Si [110], STO [001] and GaN [101] acquired at various magnifications, including 6, 8, 10, 12, 15, 20 and 25 M, were used as training data. The dwell time was 6 μs to ensure the low noise level of the AC-STEM image. All images were 2048 × 2048 pixels and the pixel length under different magnifications is labeled in Table S2. Uncorrected ADF-STEM images of Si [110], STO [001] and GaN [101] were acquired by using a JEM-F200 cold field emission electron microscope. The convergence semiangle of the probe was ∼11 mrad and the collection angles for ADF-STEM imaging were between 60 and 280 mrad. Magnification was chosen to be 20 or 25 M. All images were 2048 × 2048 pixels and the pixel length under different magnifications is labeled in Table S2. The intensity of all images displayed in this paper is normalized by dividing the maximum of the corresponding image intensity.

### SARDiffuse architecture, training and fine-tuning methods

The architecture of SARDiffuse, training methods and fine-tuning methods are included in the Supplementary information. In this study, we trained our models by fine-tuning a pretrained DDPM model, aiming to generate church images. The fine-tuning method used in this study was a LoRA method [52], which can significantly decrease the number of fine-tuning parameters, thereby improving the training and fine-tuning efficiency (see Supplementary materials for details).

### Atomic-position determination, precision test and robustness test

The atomic-position coordinates (x, y) of STEM images were calculated by using localized maximum and 2D Gaussian fitting methods [47]. As uncorrected STEM images are all noisy, a Gaussian blur was applied to ADF-STEM images before atomic-position determination.

For ADF-STEM images of Si [110], the precision can be calculated by measuring the column-to-column spacing, i.e. the a and b lattice parameters, as shown in Fig. 3a [27]. The standard deviation of this measurement is the precision of the ADF-STEM image. For measuring the column-to-column spacing, we first calculated the distances between two atoms by using the atomic coordinates and filtered out the exact a and b lattice measurements by using clustering methods.

The robustness of SARDiffuse models was evaluated by first generating 50 plausible solutions for a given input. This was achieved via changing the random seed of SARDiffuse. Then, for every atom (label i), atomic-position coordinates were calculated for the given input (${\mathrm{X}}_{\mathrm{i}}^0$, ${\mathrm{Y}}_{\mathrm{i}}^0$) and all plausible solutions (${\mathrm{X}}_{\mathrm{i}}^{\mathrm{k}}$, ${\mathrm{Y}}_{\mathrm{i}}^{\mathrm{k}}$) (*k* = 1–50). The atomic-position deviation is the difference in the atomic-position coordinates between plausible solutions and given inputs, i.e. (${\mathrm{X}}_{\mathrm{i}}^{\mathrm{k}}$ – ${\mathrm{X}}_{\mathrm{i}}^0$, ${\mathrm{Y}}_{\mathrm{i}}^{\mathrm{k}}$ – ${\mathrm{Y}}_{\mathrm{i}}^0$).

## Supplementary Material

nwaf235_Supplemental_Files
