# Supplementary material for: Deep learning for sub-ångström-resolution imaging in uncorrected scanning transmission electron microscopy
Source: Natl Sci Rev. 2025 Jun 5;12(8):nwaf235. doi: 10.1093/nsr/nwaf235 (PMC12315106; doi:10.1093/nsr/nwaf235)
Supplement: nwaf235_Supplemental_Files [file nwaf235_supplemental_files.zip › revision of Supplementary Information _NSR .docx]

*Supplementary information for*

**Deep Learning for Sub-****ångström Resolution Imaging in Uncorrected Scanning Transmission Electron Microscope**

Zanlin Qiu^1,#^, Yuan Meng^1,#^, Junxian Li^1,#^, Yanhui Hong^2,*^, Ning Li^1^, Xiaocang Han^1^, Yu Liang^1^, Wing Ni Cheng^1^,Guolin Ke^2^, Linfeng Zhang^2,3^, Weinan E^3,4,5^, Xiaoxu Zhao^1*^, Jin Zhang^1,6,7*^

^1^School of Materials Science and Engineering, Peking University, Beijing 100871, China

^2^DP Technology, Beijing 100080, China

^3^AI for Science Institute, Beijing 100084, China

^4^Center for Machine Learning Research, Peking University, Beijing 100871, China

^5^School of Mathematical Sciences, Peking University, Beijing 100871, China

^6^Center for Nanochemistry, Beijing Science and Engineering Center for Nanocarbons, Beijing National Laboratory for Molecular Sciences, College of Chemistry and Molecular Engineering, Peking University, Beijing 100871, China

^7^School of Advanced Materials, Peking University Shenzhen Graduate School, Shenzhen, Guangdong 518055, China


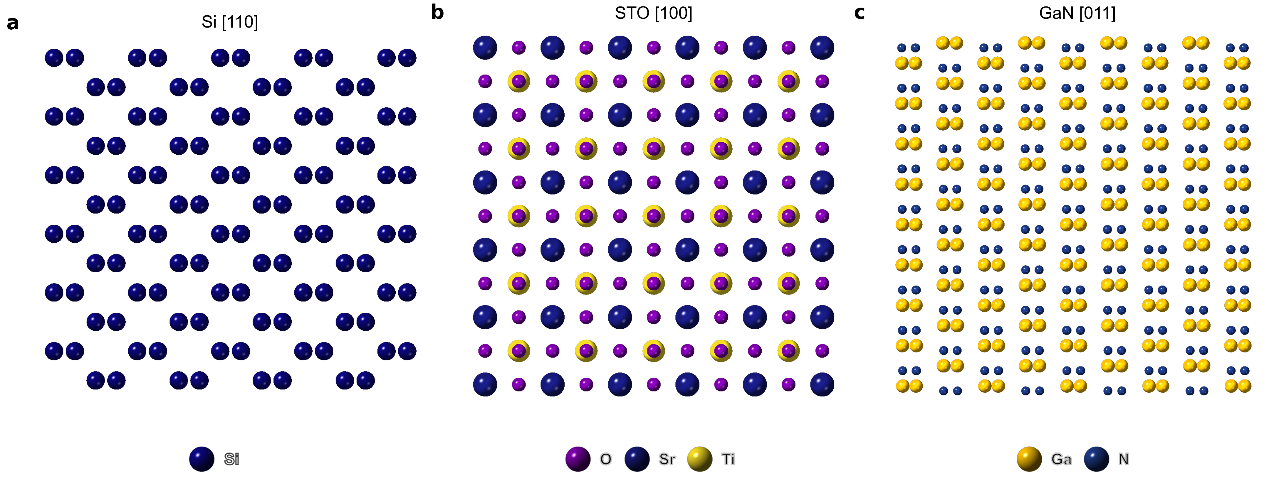


**Supplementary Fig. 1.** Atomic models of (a) silicon (Si) along the [110] axis, (b) strontium titanate (STO) along the [100] axis, and (c) GaN along the [101] axis


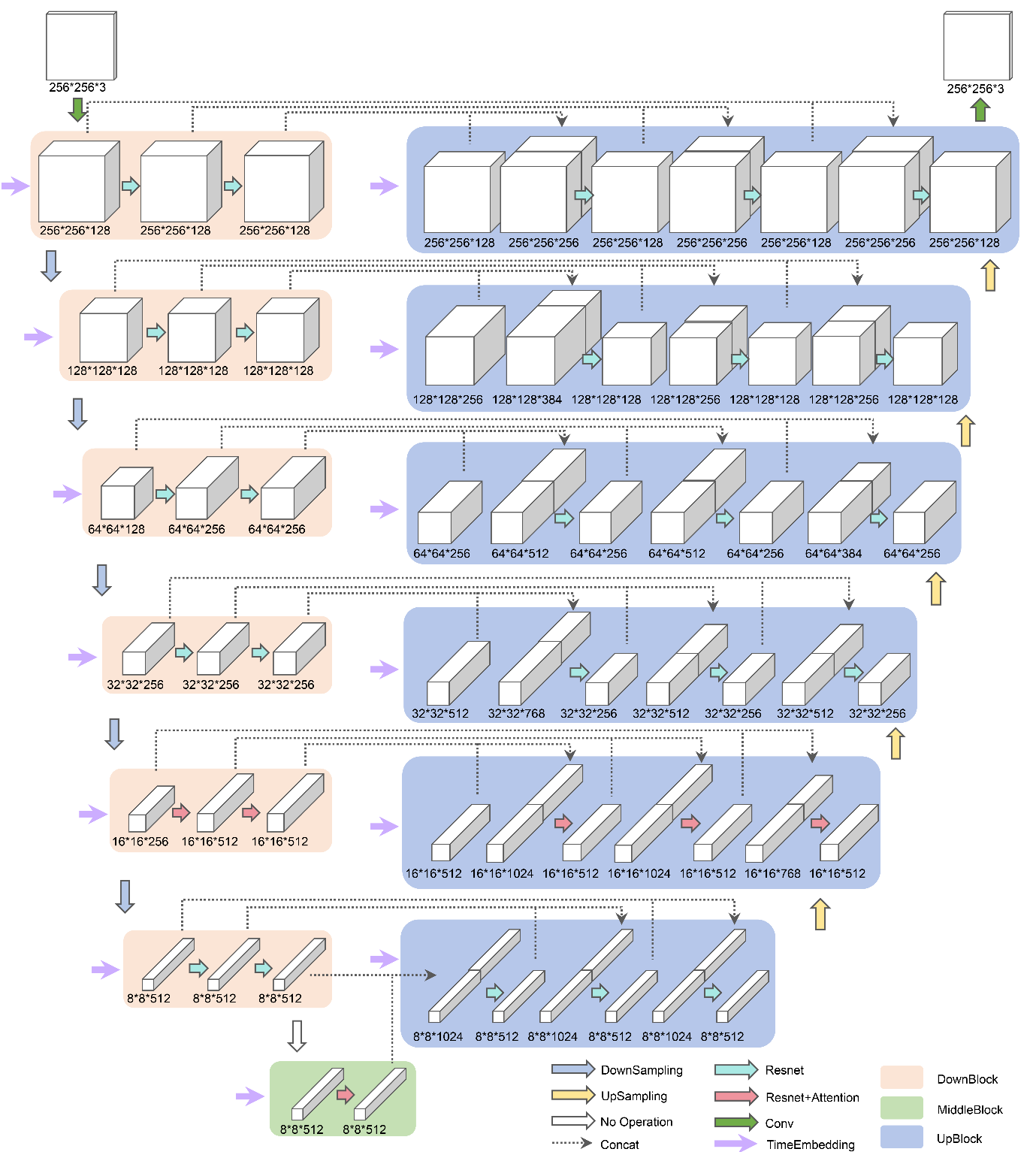


**Supplementary Fig. 2. Network architecture of the SARDiffuse model.** A traditional U-shape model involving an encoder and a decoder is employed in every step of the diffusion chain.


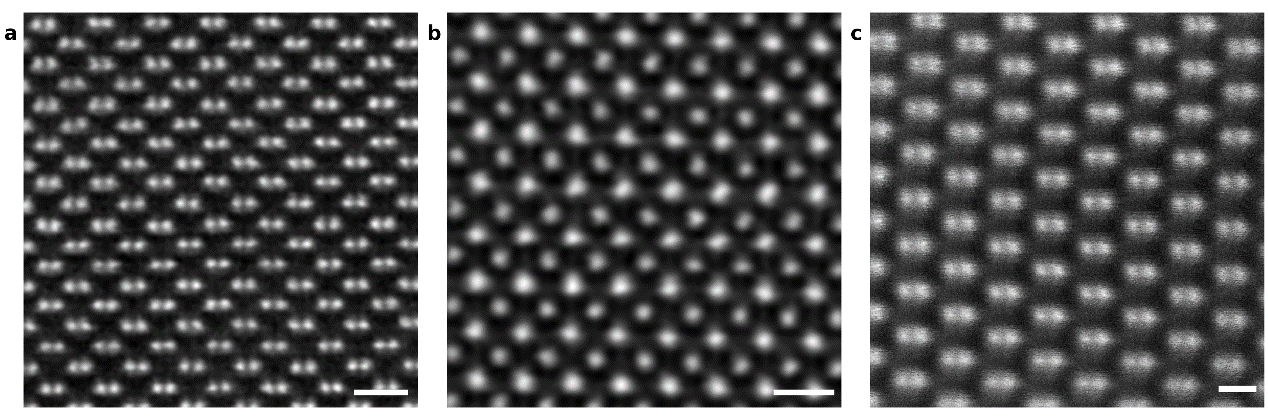


**Supplementary Fig. 3. The experimental aberration-corrected annular-dark-field scanning transmission electron microscopy (ADF-STEM) images:** (a) Si [110], (b) STO [100], and (c) GaN [101]. Scale bars, **a-b** 5 Å, **c** 2 Å.


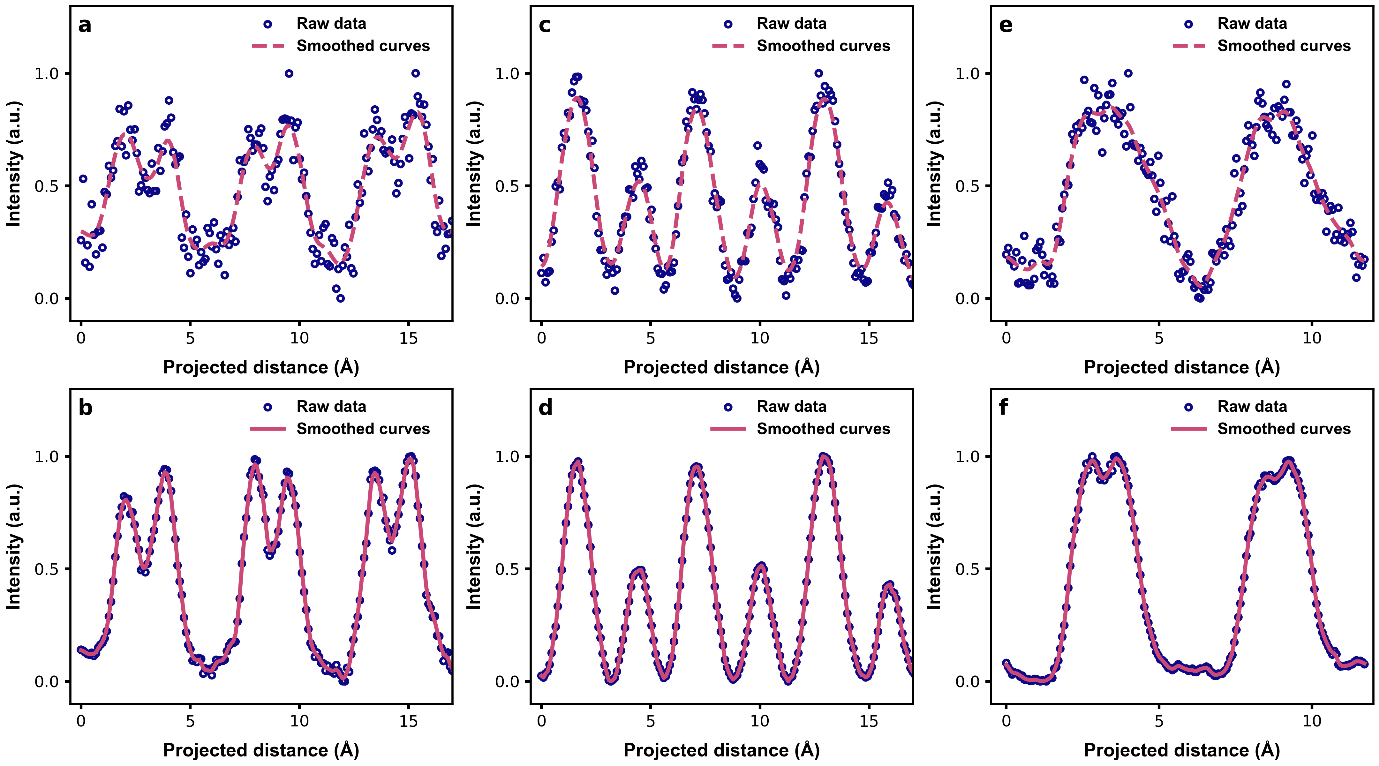


**Supplementary Fig. 4. Intensity line profiles analysis of raw (before SARDiffuse processing) and processed (after SARDiffuse processing) images. a-f** Intensity line profiles exacted from the white rectangles in **Fig. 1b-g**. The spots label the raw data and the dash lines label the data after smoothed by Gaussian Blur.


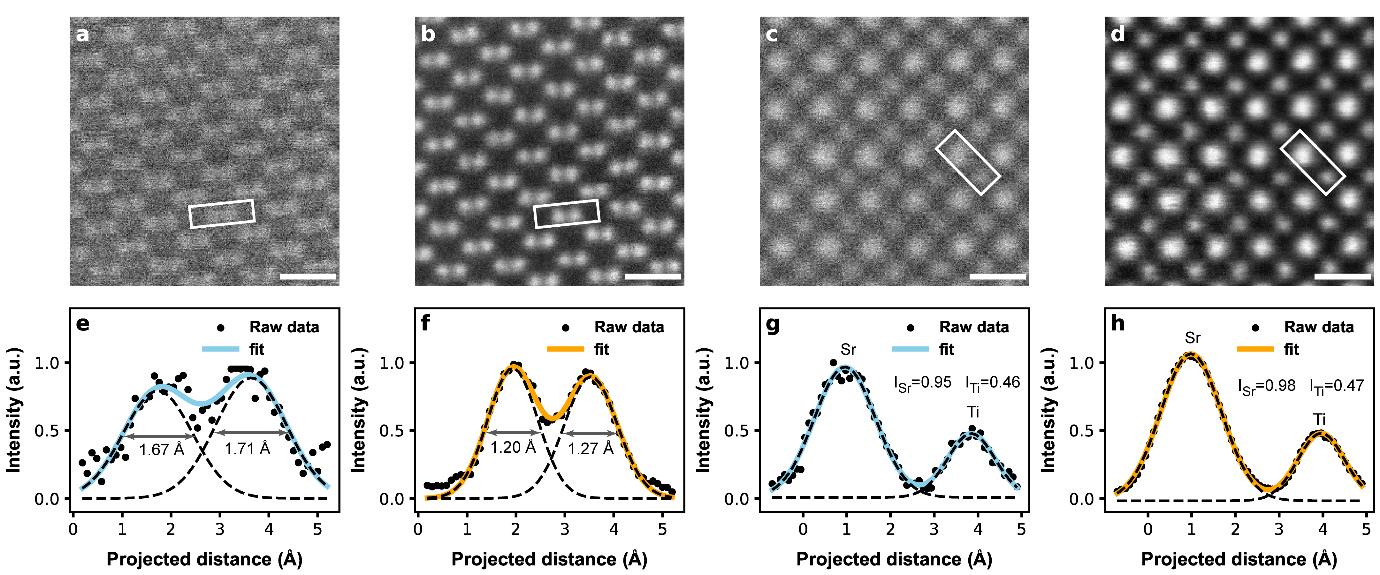


**Supplementary Fig. 5. Fitting results of the intensity line profile. a-b** Raw (a) and processed (b) ADF-STEM images of Si[110]. **c-d** Raw (c) and processed (d) ADF-STEM images of STO [001]**.** **e-h,** The intensity line profile extracted from the white rectangles in **a-d.** In **e-h**, the spots label the raw data and the color lines label the peak-fitting results with the Gaussian function. The full width at half maximum (FWHM) is marked by the grey arrows in **e** and **f**. The intensity of Sr and Ti, calculated by Gaussian fitting, are labeled in **g** and **h**. The contrast ratios of Sr and Ti ratio are calculated to be ~2.05 to ~2.08 for raw and processed images, respectively. Scale bars, 5 Å.


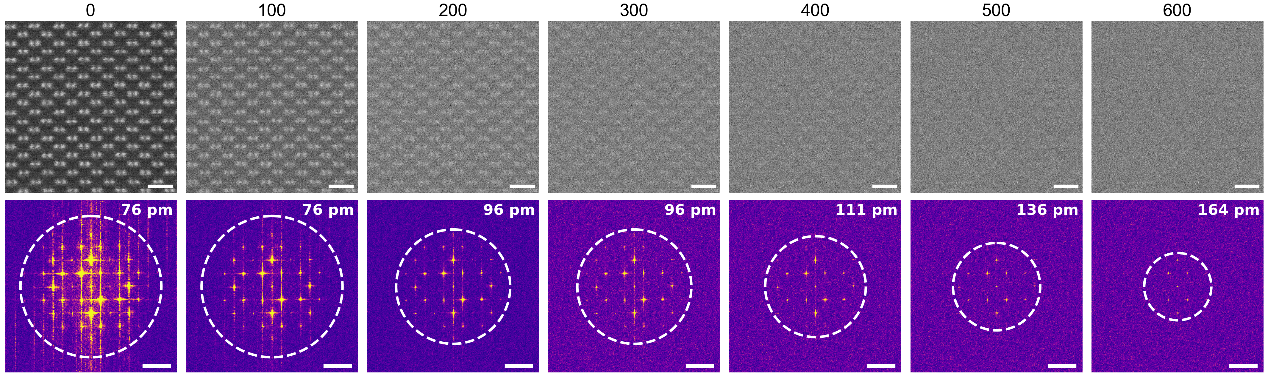


**Supplementary Fig. 6. Sequential ADF-STEM images upon a forward diffusion with increasing inference steps.** An ADF-STEM Si[110] ADF-STEM image is chosen for this evaluation. The inference steps are labled on the top of ADF-STEM images. The white circles in the fast Fourier transformation (FFT) patterns indicate the information transfer limits and the values are marked at the top right corner. Scale bars, 5 Å for images, 5 nm^-1^ for FFT power spectra


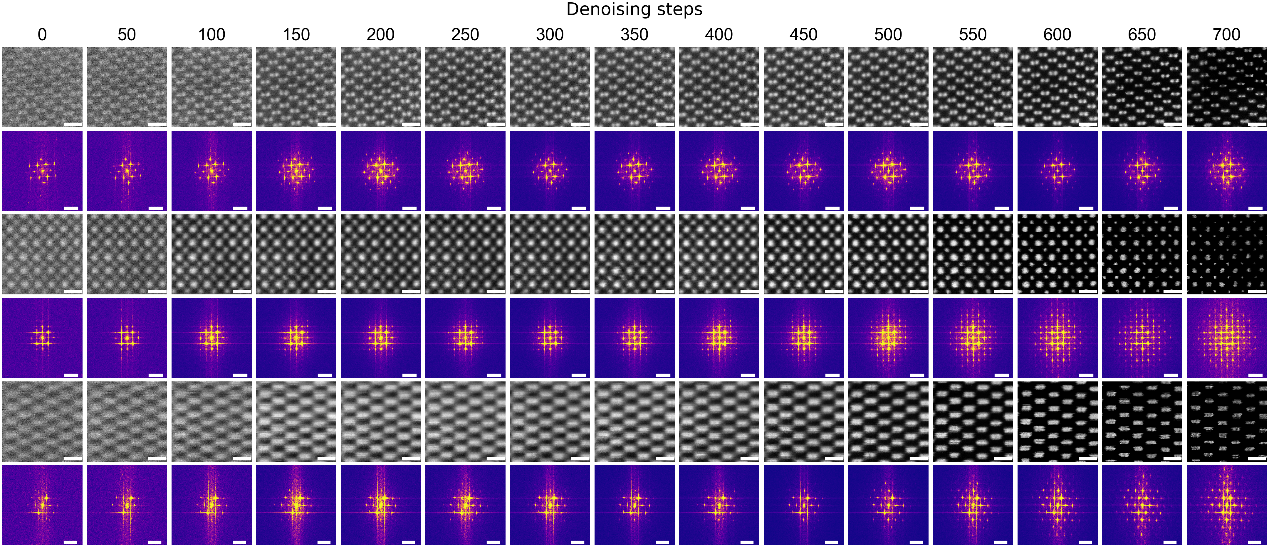


**Supplementary Fig. 7. The processed ADF-STEM images of Si[110], STO [100] and GaN [101] with various inference steps.** The inference steps consecutively increase from 0 to 700 with an increment of 50 steps. Scale bars, 5 Å for images, 5 nm^-1^ for FFT power spectra.


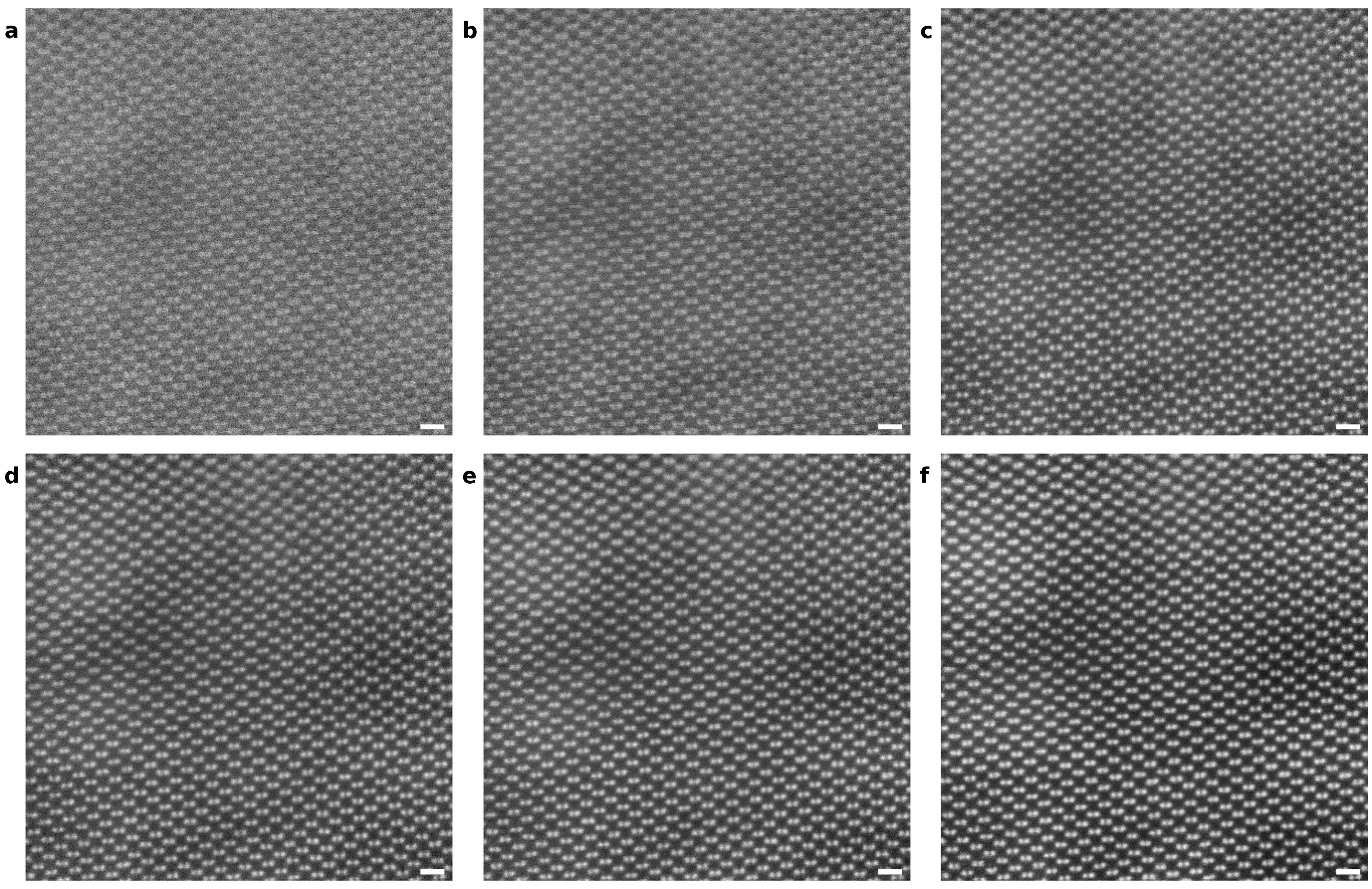


**Supplementary Fig. 8.** **Performance of SARDiffuse toward an ADF-STEM image of Si[110].** **a** The raw ADF-STEM images of Si[110] acquired by an uncorrected microscopy (JEOL F200). **b-f** The ADF-STEM images of Si[110] processed by SARDiffuse with (b) 100, (c) 200, (d) 300, (e) 400, and (f) 500 interference steps. Scale bars: 5 Å.


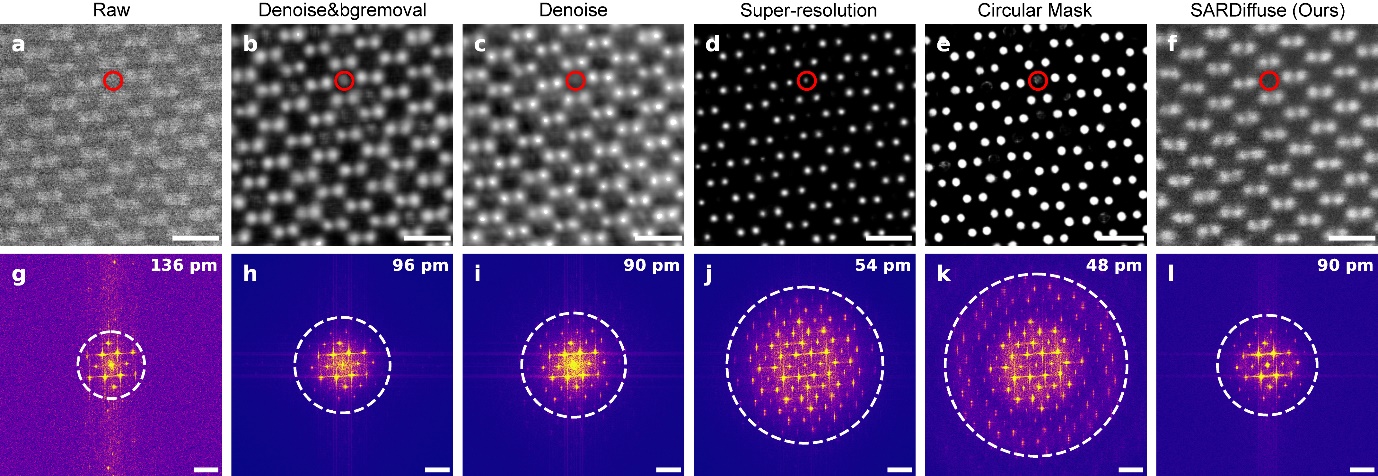


**Supplementary Fig. 9. The Comparison of performance of different modes of AtomSegNet models and SARDiffuse.** **a**. Raw STEM image of Si [110] acquired by an uncorrected microscopy (JEOL F200). **b-e** Images after processed by different approaches, including denoise and background subtracted (Denoise&bgremoval) (b), Denoise (c), Super-resolution (d) and Circular mask segmentation (Circular Mask) (e) methods. **f** Image processed by SARDiffuse. **g-l** corresponding FFT pattern of **a-f**. Scale bars, **a-f** 5 Å; **g-l** 5 nm^-1^.


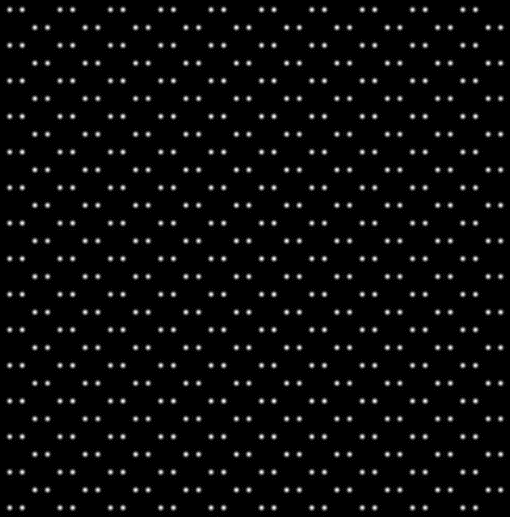

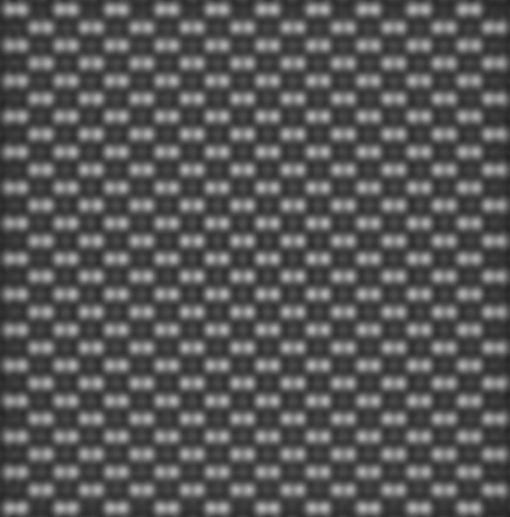

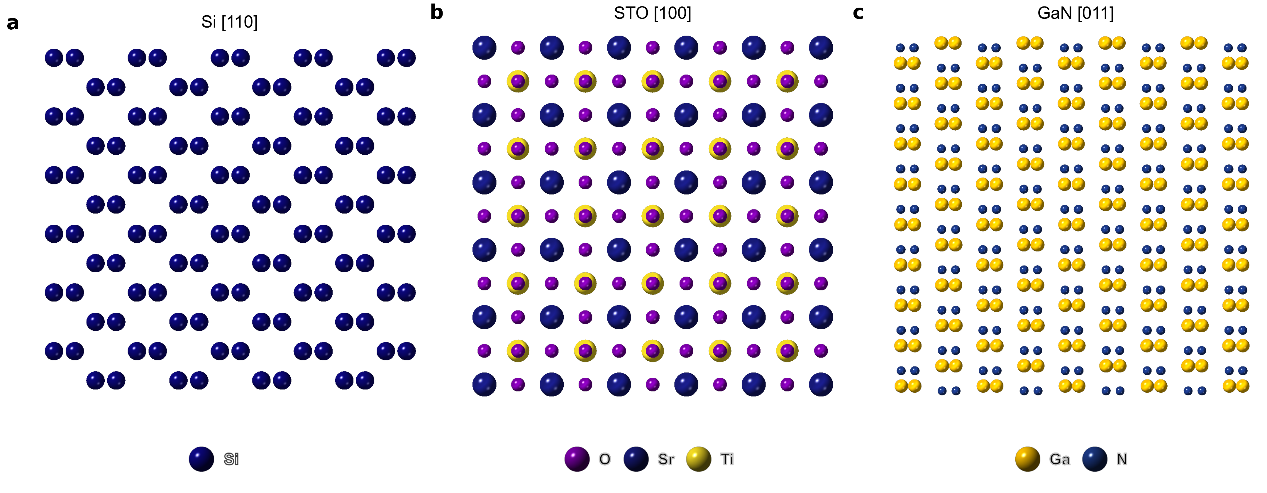

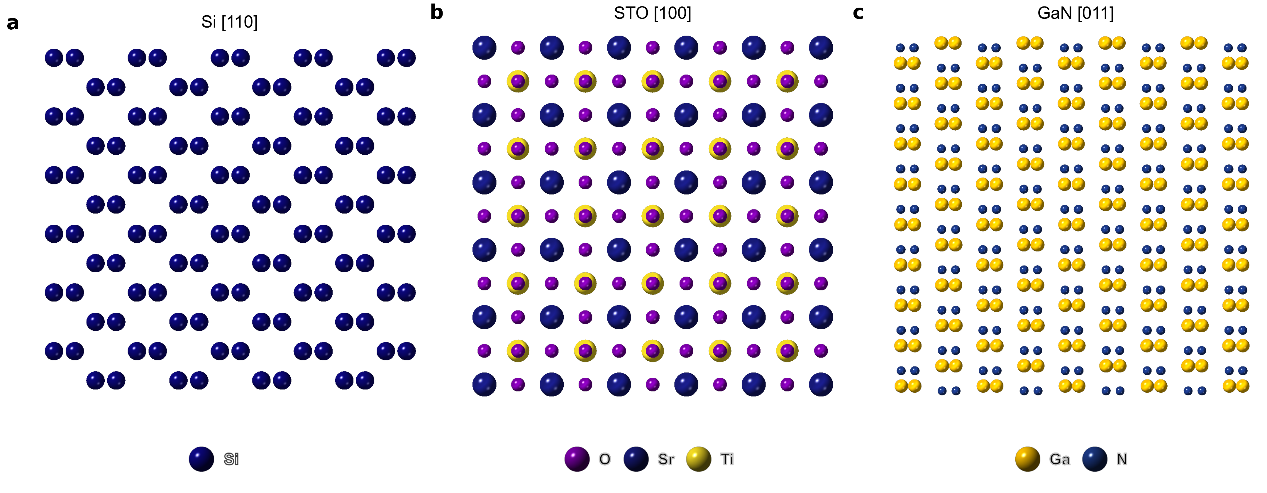


**Supplementary Fig. 10. STEM Simulation of ADF-STEM images of Si[110]. a-b** simulated ADF-STEM images of Si[110] with (a) C_3_= 0 mm (b) C_3_ = 0.5 mm. The acceleration voltage, converge angle and collection angles are 200 kV, 24 mrad and 80-250 mrads, respectively. The simulation parameters are exclusively the same except that the C_3_ is chosen to be 0.5 mm in (b) with compensated defocus to be at the Scherzer defocus. Scale bars, 5 Å.


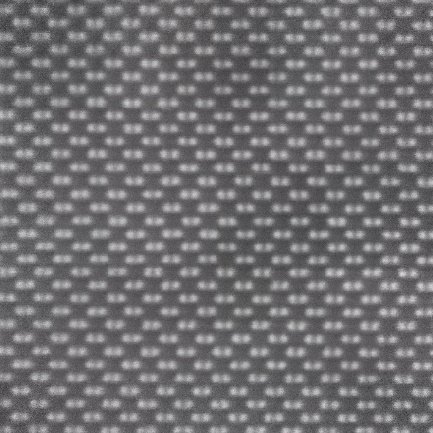

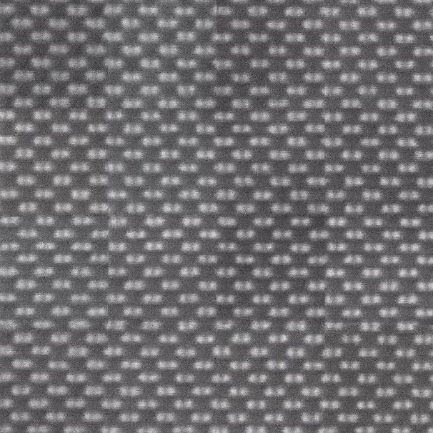


Stitched lines


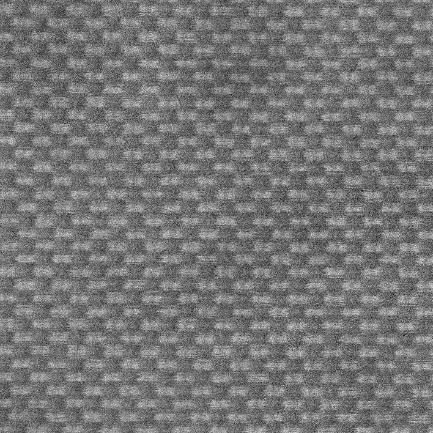

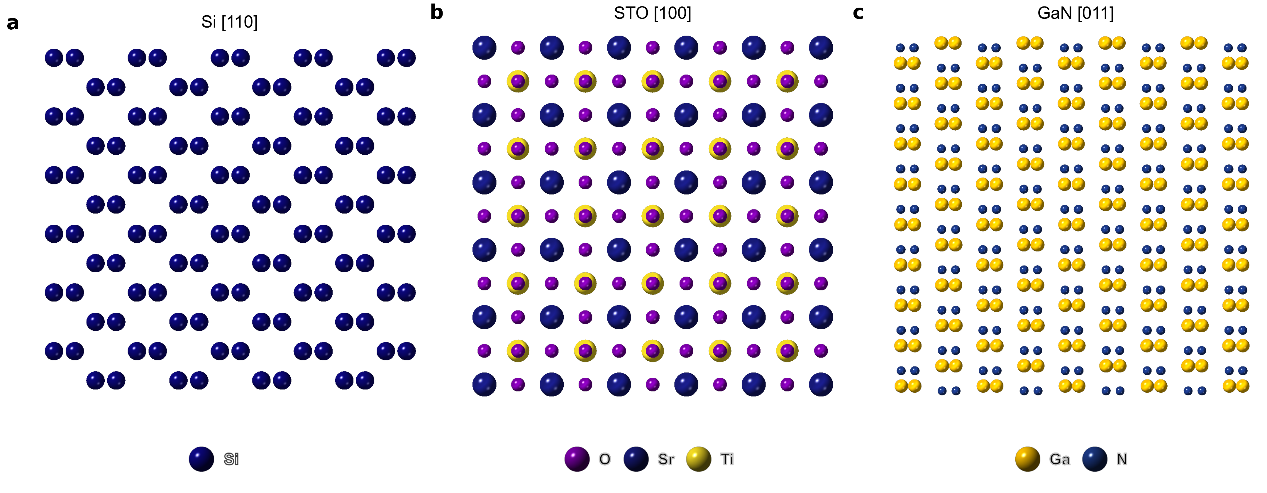

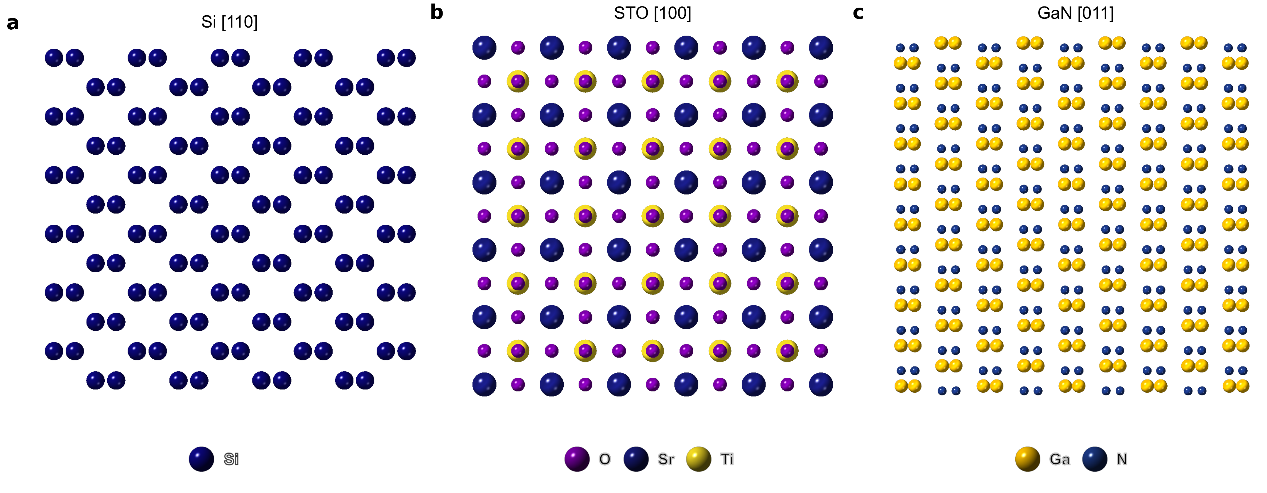

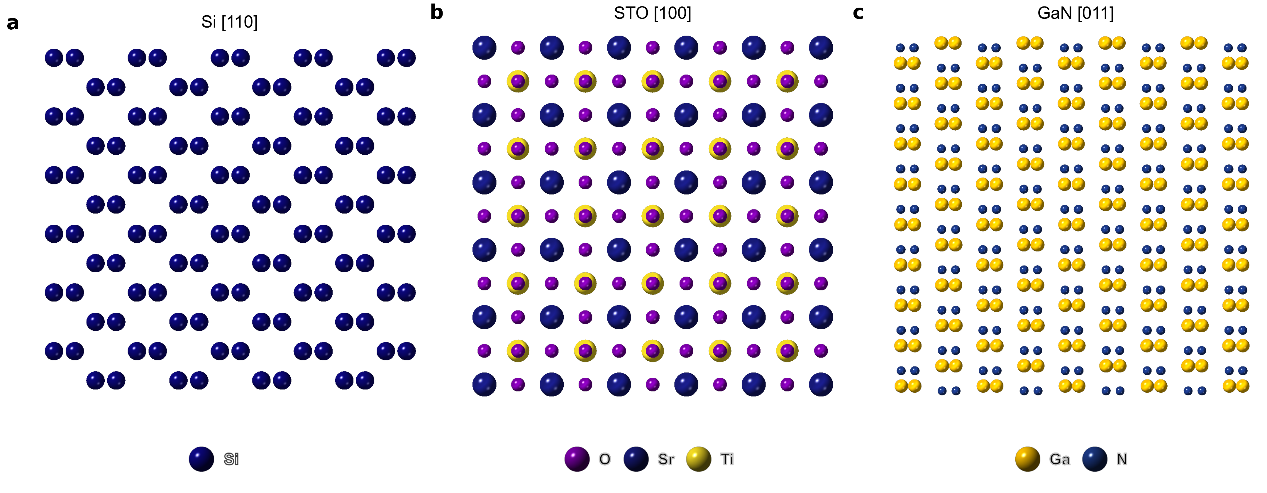


S**upplementary Fig. 11. Comparision of performance of diferente stiching algorithms. a** Processed ADF-STEM images of Si [110] stitched by average overlapping. **b** Processed image stitched using weighted averaging algorithm. **c** The raw uncorrected Si [110] ADF-STEM images. Stride parameters used during stitching for (a) and (b) are all 128 pixels. The red arrow labels the artifacts, *i.e.* stitching lines. The red dash lines show the drifting information. Scale bars: 5 Å.


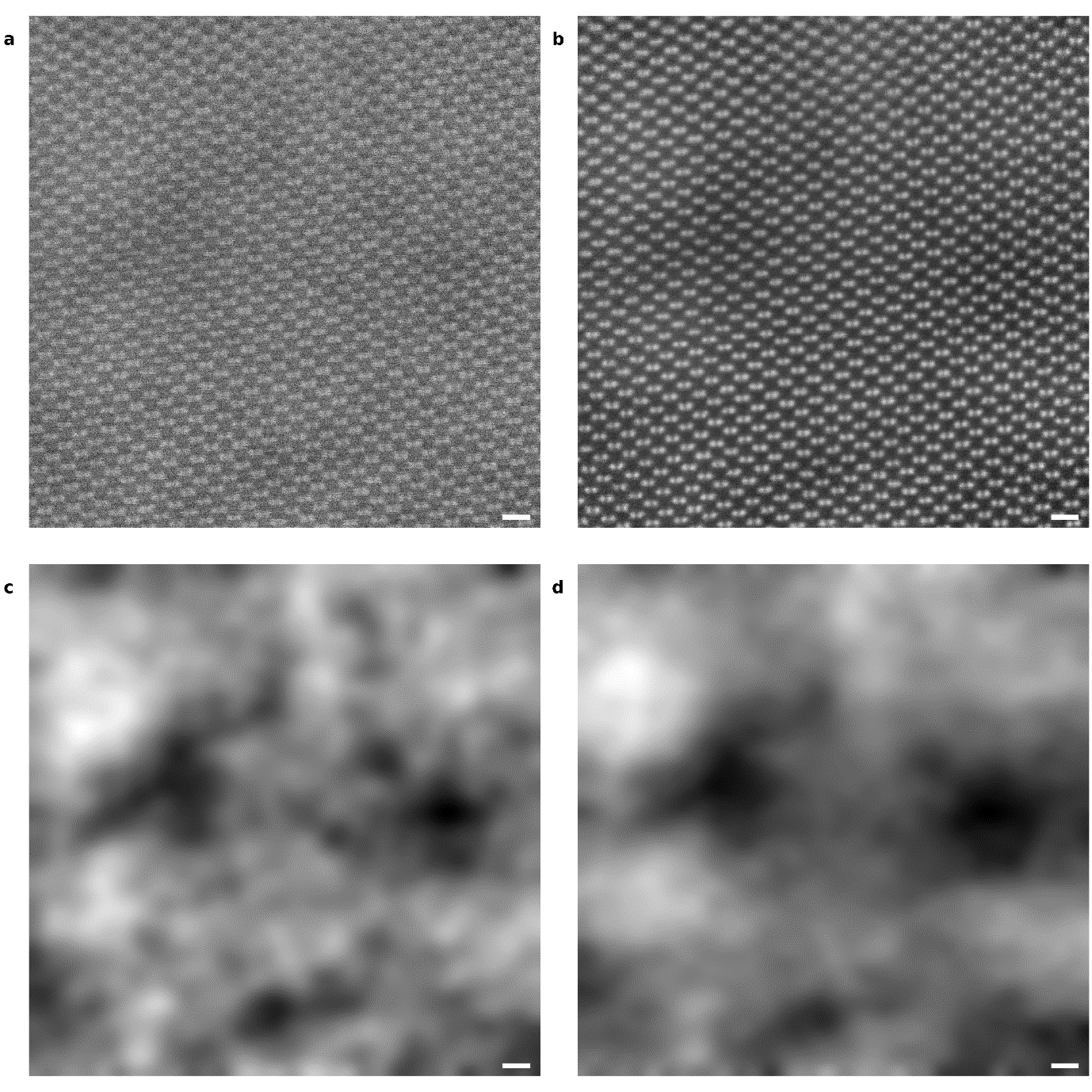


**Supplementary Fig. 12. Background analysis of raw and processed ADF-STEM images. a** The raw ADF-STEM images of Si[110], which is same as supplementary Fig. 6a. **b** The ADF-STEM images of Si[110] processed by SARDiffuse with 350 inference steps. **c-d** Background information of (a) and (b), respectively. The background information is obtained by doing Gaussian blurring. To extract the background information, the standard deviation of the Gaussian keneral used during blurring is 20. **c** is almost analogous to **d**, suggesting that SARDiffuse effectively preserve the background information. Scale bars: 5 Å.

**Supplementary** **Table 1. The average deviation of atomic column positions**

| Denoising Steps | 100 | 200 | 300 | 400 | 500 |
| --- | --- | --- | --- | --- | --- |
| Deviation (pixel) | 1.008±0.452 | 1.008±0.452 | 1.539±0.500 | 1.712±0.599 | 1.876±0.740 |
| Deviation along X (pixel) | 0.480±0.266 | 0.480±0.266 | 0.767±0.382 | 0.840±0.458 | 0.976±0.577 |
| Deviation along Y (pixel) | 0.729±0.419 | 0.729±0.419 | 1.095±0.532 | 1.259±0.611 | 1.344±0.733 |

Note: for the input image, the pixel size is 9.5 pm.

**Supplementary** **Table 2.** **The average deviation of atomic column positions**

| Magnification | 6M | 8 M | 10 M | 12 M | 15 M | 20 M | 25 M |
| --- | --- | --- | --- | --- | --- | --- | --- |
| Pixel Length (pm) | 16.4 | 12.25 | 9.8 | 7.84 | 6.5 | 4.9 | 3.25 |

**Supplementary section 1: SARDiffuse architecture and training objective**

SARDiffuse is based on the Denoising Diffusion Probabilistic Model (DDPM), involving a forward process and a reverse process. The forward diffusion process converts the target data distribution (e.g., the real image distribution) into a tractable probability distribution (e.g., the Gaussian distribution) by gradually adding noise to the aberration-corrected scanning transmission electron microscopy (AC-STEM) image with random steps (0-1000). DDPM then learns to restore AC-STEM images from noisy images by eliminating noise at each step, thereby reversing the forward diffusion process^1-4^. In the following section, we will elaborate on the fundamental basis of both processes.

**1.1 Forward** **diffusion process**

The forward diffusion process continuously adds Gaussian noise to the given initial data $\mathbf{x}_{0}$ following the Markov process, producing noisy data $\mathbf{x}_{1}$**,** $\mathbf{x}_{2}$**, … ,** $\mathbf{x}_{T}$, as described by equation 1^1-4^.

$\boldsymbol{q}\left( \mathbf{x}_{t} | \mathbf{x}_{t-1} \right)\boldsymbol{=N}\left( \mathbf{x}_{t}\boldsymbol{;}\sqrt{1-\beta_{t}}\mathbf{x}_{t-1},\beta_{t}\boldsymbol{\varepsilon} \right)$ $\boldsymbol{q}\left( \mathbf{x}_{\mathbf{1:T}} \right)\boldsymbol{=}\prod_{\boldsymbol{t=1}}^{\boldsymbol{T}} \boldsymbol{q}\left( \mathbf{x}_{t} | \mathbf{x}_{t-1} \right)$ **(1)**

Where $\mathbf{x}_{\mathbf{t}}$ denotes the noisy data at step t. $\boldsymbol{\varepsilon}$ is the standard Gaussian noise. $\beta_{t}\in\left( 0,1 \right)$ is the noise variance schedule. $\boldsymbol{N}\left( \mathbf{x}_{t}\boldsymbol{;}\sqrt{1-\beta_{t}}\mathbf{x}_{t-1},\beta_{t}\boldsymbol{\varepsilon} \right)$ is equivalent to $\mathbf{x}_{t}\boldsymbol{=}\sqrt{1-\beta_{t}}\mathbf{x}_{t-1}+\beta_{t}\boldsymbol{\varepsilon}$.

Based on the Markov chains hypothesis, the above process is equivalent to the sample $\mathbf{x}_{t}$ at an arbitrary step 𝑡 from $\mathbf{x}_{0}$^1-4^.

$\boldsymbol{q}\left( \mathbf{x}_{t} | \mathbf{x}_{0} \right)\boldsymbol{=N}\left( \mathbf{x}_{t}\boldsymbol{;}\sqrt{\bar{\alpha_{t}}}\mathbf{x}_{0},\left( 1-\bar{\alpha_{t}} \right)\boldsymbol{\varepsilon} \right)$ **(2)**

Where $\alpha_{t}=1-\beta_{t}$ and $\bar{\alpha_{t}}=\prod_{i=1}^{t} \alpha_{i}$

As suggested by equation (2), the $\mathbf{x}_{\mathbf{t}}$ will converge to a pure Gaussian distribution when t is sufficiently large enough. Usually, $\beta_{t}$ is a small number close to 0 and can be learned by reparameterization or held constant as hyperparameters. In this study, we held $\beta_{t}$ as the constant to simplify the forward diffusion process.

**1.2 Reverse process:**

The reverse process uses a parametric neural network model with parameters 𝜃 to invert the diffusion process and generates $\mathbf{x}_{\mathbf{0}}$ from pure Gaussian random noise $\mathbf{x}_{\mathbf{t}}$^1-4^.

$\boldsymbol{p}_{\theta}\left( \mathbf{x}_{t-1} | \mathbf{x}_{t} \right)\boldsymbol{=N}\left( \mathbf{x}_{t-1}\boldsymbol{;}\boldsymbol{\mu}_{\theta}\left( \mathbf{x}_{t}\boldsymbol{,}t \right),\boldsymbol{\beta}_{t}\mathbf{I} \right)$ **(3)**

Where $\boldsymbol{\mu}_{\theta}\left( \mathbf{x}_{t}\boldsymbol{,}t \right)$ and $\boldsymbol{\beta}_{t}$ are denoising functions realized by a deep neural network with parameters 𝜃 to predict the mean and covariance matrix and 𝑡 is the reverse step index. $\mathbf{I}$ is random noise. The addition of random noise term is for high performance of the model^1-4^.

Training is performed by minimizing the variational lower bound on negative log likelyhood^1-4^:

$\mathcal{L}_{VLB}=E_{q(\boldsymbol{x}_{0})}[-\log\frac{\boldsymbol{p}_{\theta}\left( \boldsymbol{x}_{0:T} \right)}{\boldsymbol{q}\left( \mathbf{x}_{1:T} | \boldsymbol{x}_{0} \right)}]$ **(4)**

After minimizing $\mathcal{L}_{VLB}$, the parameter $\theta$ can be determined. However, equation (4) is not a good way to optimize and thus Ref ^1^ proposed to use an equivalent simple form as loss function of training as suggested by equation (5).

$L_{simple}\left( \theta\right)=E_{t,\boldsymbol{x}_{0},\varepsilon}[\left\| \boldsymbol{\varepsilon}-\boldsymbol{\varepsilon}_{\theta} \left( \mathbf{x}_{\mathbf{t}}\boldsymbol{,} t \right) \right\|^{2}]=E_{t,\boldsymbol{x}_{0},\varepsilon}[\left\| \boldsymbol{\varepsilon}-\boldsymbol{\varepsilon}_{\boldsymbol{\theta}}\left( \sqrt{\bar{\alpha_{t}}}\mathbf{x}_{0}+\left( 1-\bar{\alpha_{t}} \right)\boldsymbol{\varepsilon} \right) \right\|^{2}]$ **(5)**

After $\theta$ is determined, based on the Gaussian noise addition of the forward process and Bayesian theorem, the parameter $\boldsymbol{\mu}_{\theta}\left( \mathbf{x}_{t}\boldsymbol{,}t \right)$ and $\boldsymbol{\beta}_{t}$ can be expressed as equation (6) ^1-4^.

$\left\{ \begin{aligned} \boldsymbol{\mu}_{\theta}\left( \mathbf{x}_{t}\boldsymbol{,}t \right)=\frac{1}{\sqrt{\alpha_{t}}}\left( \mathbf{x}_{\mathbf{t}}\boldsymbol{-}\frac{1\boldsymbol{-}\alpha_{t}}{\sqrt{1\boldsymbol{-}\bar{\alpha_{t}}}}\boldsymbol{\varepsilon}_{\theta} \left( \mathbf{x}_{\mathbf{t}}\boldsymbol{,} t \right) \right) \\ \boldsymbol{\beta}_{t}=\frac{1\boldsymbol{-}\bar{\alpha_{t-1}}}{1\boldsymbol{-}\bar{\alpha_{t}}} \end{aligned} \right.$ **(6)**

**1.3. Training**

In this study, we train SARDiffuse by fine-tuning a pre-trained DDPM model aiming to generate church images (https://huggingface.co/google/ddpm-church-256). The fine-tuning method used in this study is a low-rank adaptation (LoRA) method^5^, which can significantly decrease the number of fine-tuning parameters, thereby improving the training and fine-tuning efficiency (see supplementary section 2). For the sake of training efficiency and requirements of pre-trained DDPM models, we cropped 2048 × 2048 AC-STEM images to 256 × 256 image patches with a stride parameter of 128 for training, resulting in around 13,000 patches for training. We observed that the performance of the DDPM model is sensitive to the field of view (FOV) and pixel length of STEM images. To overcome this issue, experimental AC-STEM images with different magnifications as well as the pixel length (Supplementary Table 2) are acquired and used as training data.

**1.4 Inference stage and stitching algorithms**

In the inference stage, one raw image acquired at an uncorrected STEM was first cropped to 256 × 256 image patches with the stride parameter of 128. Then, the corresponding output patches from the SARDiffuse model were stitched together using weighted averaging stitching methods. In this stitching method, each predicted patch is placed back into its original position according to a weighting scheme based on the distance from the center of each patch (see supplementary section 3 for more details). Pixels nearer to the center are assigned higher weights, while those closer to the edges receive lower weights. In regions where patches overlap, we compute the weight average of the predictions to mitigate any discrepancies (see supplementary section 3 for more details).

**Supplementary section 2:** Low-rank adaptation fine-tuning method

The SARDiffuse model is fine-tuned by low-rank adaptation (LoRA) methods ^5^. Unlike traditional fine-tuning methods that update all the parameters of the model, LoRa introduces low-rank approximations to the weight updates of certain layers, particularly the query and value projection matrices in the self-attention mechanism. This can largely reduce the number of parameters and resource demands of fine-tuning tasks. Here is a specific introduction of LoRa methods:

Let $W_{0}\in R^{d\times k}$ be a pre-trained weight matrix (d and k are the weight matrix size). Instead of updating $W_{0}$ directly during fine-tuning, LoRa models the weight update $\Delta W$ as a low-rank decomposition:

$\Delta W=AB$ **(7)**

where $A\in R^{d\times r}$ and $B\in R^{r\times k}$, and $r\ll min(d, k)$ is the rank of the decomposition. During fine-tuning, only $A$ and $B$ are updated, while $W_{0}$ remains frozen. The forward pass computation becomes:

$h=W_{0}x+ \Delta Wx=W_{0}x+A(Bx)$ **(8)**

where $x$ is the input, and $h$ is the output of the layer.

Based on low-rank matrices, the number of trainable parameters is significantly reduced. The total number of parameters for $A$ and $B$ is:

$Total Parameters=r\times(d+k)$ **(9)**

Compared to the original weight matrix $W_{0}$ with $d\times k$ parameters, LoRA requires much fewer parameters when $r$ is small.

LoRA introduces minimal additional computational overhead. The matrix multiplication $A(Bx)$ is efficient due to the low rank $r$. Moreover, since $W_{0}$ is fixed, it can be cached or quantized to further improve efficiency.

**Supplementary section 3: Stitching algorithm**

For the sake of training efficiency and requirements of pre-trained DDPM models, SARDiffuse is trained by 256 × 256 pixels images patches of AC-STEM images (See methods for more details). This requires that the input of the inference stage should also have dimensions of 256 × 256 pixels. However, STEM images usually have dimensions of 512 × 512 pixels, 1024 × 1024 pixels, or 2048 × 2048 pixels. To address this issue, we crop STEM images into 256 × 256 pixel patches, processed individually by SARDiffuse, and subsequently stitched together. Generally, there are two stitching methods implemented to achieve this goal: averaging overlapping method and weighted averaging. The following parts will introduce the two stitching algorithms in detail.

- 1. **Averaging overlapping**

In this method, each predicted patch is placed back into its original position within the image, determined by the stride parameters $(s_{x}, s_{y})$. In regions where patches overlap, we compute the average of the predictions to mitigate any discrepancies. The steps are:

1. Initialization: Create a zero-initialized matrix $final\_img$ of dimensions $(N_{x}, N_{y})$. Additionally, initialize a count matrix $cnt$ of the same dimensions to keep track of the accumulation.
2. Accumulation of Predictions: For each predicted patch ${pred}_{i,j}$, compute the starting positions $x_{i}$ and $y_{j}$:

$x_{i}=x_{list}\left[ i \right], y_{j}=y_{list}\left[ j \right]$, **(10)**

where

$$x_{list}=\left\{ \begin{aligned} \left\{ 0,s_{x}, 2s_{x}, \ldots, \left\lfloor\frac{N_{x}-l_{x}}{s_{x}} \right\rfloor s_{x} \right\}, if \left\lfloor\frac{N_{x}-l_{x}}{s_{x}} \right\rfloor s_{x}=N_{x}-l_{x}, \\ \left\{ 0,s_{x}, 2s_{x}, \ldots, \left\lfloor\frac{N_{x}-l_{x}}{s_{x}} \right\rfloor s_{x}, N_{x}-l_{x} \right\}, otherwise. \end{aligned} \right.$$

$$y_{list}=\left\{ \begin{aligned} \left\{ 0,s_{y}, 2s_{y}, \ldots, \left\lfloor\frac{N_{y}-l_{y}}{s_{y}} \right\rfloor s_{y} \right\}, if \left\lfloor\frac{N_{y}-l_{y}}{s_{y}} \right\rfloor s_{y}=N_{y}-l_{y}, \\ \left\{ 0,s_{y}, 2s_{y}, \ldots, \left\lfloor\frac{N_{y}-l_{y}}{s_{y}} \right\rfloor s_{y}, N_{y}-l_{y} \right\}, otherwise. \end{aligned} \right.$$

Update $final\_img$ and $cnt$ as follows:

$image\left[ x_{i}:x_{i}+l_{x},y_{j}:y_{j}+l_{y} \right]+={pred}_{i,j}$,

$cnt\left[ x_{i}:x_{i}+l_{x},y_{j}:y_{j}+l_{y} \right]+=1$.

1. Calculation of the Average: Compute the final stitched image by dividing the accumulated pixel values by the corresponding counts:

$$output=\frac{final\_img}{cnt}$$

Finally, The stitching process can be formalized as:

$output\left( p,q \right)=\frac{\sum_{i,j} \delta_{\left( p,q \right)\in R_{i,j}}\cdot{pred}_{i,j}(p-x_{i}, q-y_{j})}{\sum_{i,j} \delta_{\left( p,q \right)\in R_{i,j}}}$ **(11)**

where $\left( p,q \right)$ represents the pixel coordinates in the original image; $R_{i,j}$ is the spatial region occupied by the patch ${pred}_{i,j}$, starting at $(x_{i}, y_{j})$; $x_{i}=x_{list}\left[ i \right], y_{j}=y_{list}\left[ j \right]$; $\delta_{\left( p,q \right)\in R_{i,j}}$ is the indicator function:

$$\delta=\left\{ \begin{aligned} 1, if \left( p,q \right)\in\left[ x_{i}:x_{i}+l_{x} \right)\times\left[ y_{j}:y_{j}+l_{y} \right), \\ 0, otherwise. \end{aligned} \right.$$

- 1. **Weighted Averaging with Distance Coefficients**

The second method enhances the first by incorporating a weighting scheme based on the distance from the center of each patch. The stride parameters $(s_{x}, s_{y})$ influence the degree of overlap and, consequently, the regions where the weights are applied. Pixels nearer to the center are assigned higher weights, while those closer to the edges receive lower weights. This approach reduces edge artifacts and improves the overall quality of the stitched image.

1. Generation of the Distance Coefficient Matrix: For each patch, generate a weight matrix $w$ where the weights decrease linearly from the center to the edges. The minimum weight at the edges is specified by $min\_value$.

$w\left( p^{'},q^{'} \right)=1-(\frac{d\left( p^{'},q^{'} \right)}{D_{max}})(1-min\_value)$ **(12)**

with $\left( p^{'},q^{'} \right)$ being the coordinates within the patch ${pred}_{i,j}$, where $\left( p',q' \right)\in\left[ 0:l_{x} \right)\times\left[ 0:l_{y} \right)$; $d\left( p^{'},q^{'} \right)$ representing the Euclidean distance from the center of the patch:

$d\left( p^{'},q^{'} \right)=\sqrt{{(p^{'}-\frac{l_{x}-1}{2})}^{2}+{(q^{'}-\frac{l_{y}-1}{2})}^{2}}$ **(13)**

$D_{max}$ being the maximum distance from the center to a corner of the patch:

$D_{max}=\sqrt{{(\frac{l_{x}-1}{2})}^{2}+{(\frac{l_{y}-1}{2})}^{2}}$ **(14)**

1. Weighted Accumulation of Predictions: For each predicted patch ${pred}_{i,j}$, compute the starting positions $x_{i}$ and $y_{j}$ :

$x_{i}=x_{list}\left[ i \right], y_{j}=y_{list}\left[ j \right]$,

where

$$x_{list}=\left\{ \begin{aligned} \left\{ 0,s_{x}, 2s_{x}, \ldots, \left\lfloor\frac{N_{x}-l_{x}}{s_{x}} \right\rfloor s_{x} \right\}, if \left\lfloor\frac{N_{x}-l_{x}}{s_{x}} \right\rfloor s_{x}=N_{x}-l_{x}, \\ \left\{ 0,s_{x}, 2s_{x}, \ldots, \left\lfloor\frac{N_{x}-l_{x}}{s_{x}} \right\rfloor s_{x}, N_{x}-l_{x} \right\}, otherwise. \end{aligned} \right.$$

$$y_{list}=\left\{ \begin{aligned} \left\{ 0,s_{y}, 2s_{y}, \ldots, \left\lfloor\frac{N_{y}-l_{y}}{s_{y}} \right\rfloor s_{y} \right\}, if \left\lfloor\frac{N_{y}-l_{y}}{s_{y}} \right\rfloor s_{y}=N_{y}-l_{y}, \\ \left\{ 0,s_{y}, 2s_{y}, \ldots, \left\lfloor\frac{N_{y}-l_{y}}{s_{y}} \right\rfloor s_{y}, N_{y}-l_{y} \right\}, otherwise. \end{aligned} \right.$$

Update $final\_img$ and $cnt$ using the weighted predictions:

$image\left[ x_{i}:x_{i}+l_{x},y_{j}:y_{j}+l_{y} \right]+={pred}_{i,j}\times w$,

$cnt\left[ x_{i}:x_{i}+l_{x},y_{j}:y_{j}+l_{y} \right]+=w$.

1. Calculation of the Weighted Average: Obtain the final stitched image:

$$output=\frac{final\_img}{cnt}$$

Finally, the weighted stitching process is mathematically represented as:

$output\left( p,q \right)=\frac{\sum_{i,j} \delta_{\left( p,q \right)\in R_{i,j}}\cdot{w(p-x_{i}, q-y_{j})\cdot pred}_{i,j}(p-x_{i}, q-y_{j})}{\sum_{i,j} \delta_{\left( p,q \right)\in R_{i,j}}\cdot w(p-x_{i}, q-y_{j})}$ **(15)**

The weighted averaging technique mitigates the influence of less accurate predictions typically found at the edges of patches. Since models often exhibit higher predictive accuracy near the center of patches, assigning greater weights to central pixels enhances the fidelity of the stitched image. The stride parameters $(s_{x}, s_{y})$ affect the degree of overlap, and using weights helps to smooth transitions between overlapping regions, especially when strides are less than the patch size.

**3.3 Comparision of two stitching algorithms**

Both averaging overlapping and weight averaging methods have been tested in this study. Averaging overlapping generates edge artifacts (stitched lines) during stitching (Supplementary Fig. 11a) while the weighted averaging avoids the formation of edge artifacts (Supplementary Fig. 11b). These artifacts might result in the distortion of atoms in ADF-STEM images (Supplementary Fig. 11a), thus impeding the precise atomic position determination.

**Supplementary section 4: Inference steps**

The super-resolution ability of SARDiffuse can be controlled by the inference steps (Fig 1a and Fig 2). Introducing additional inference steps will result in improved information transfer limits and lower noise levels. Increasing inference steps to more than 500 steps can further improve the spatial resolution but the processed images might be untrusted as some atom blobs in the images are diminished (supplementary Fig. 7). Thus, the inference steps should be carefully optimized.

However, determining the optimal number of inference steps is challenging. For example, for uncorrected Si [110] ADF-STEM images, processed images with 400 and 450 steps are nearly identical (Supplementary Fig. 7). This suggests that defining a fixed "optimized" number of inference steps is not meaningful. Instead, we provide several criteria for determining the range of valid inference steps. All the corresponding codes are available in <https://github.com/dptech-corp/SARDiffuse>.

The minimum number of inference steps can be determined by calculating the information transfer limit. Typically, sub-ångström resolution (< 100 pm) is desired, and there is a minimum number of inference steps required to achieve sub-ångström resolution (Fig. 2 and Supplementary Fig. 7).

The maximum number of inference steps can be determined in several methods. One approach is to define a threshold for the permissible deviation based on the user's requirements (Fig. 3). For instance, if the user prefers an atomic deviation within 1.7 pixels, 400 steps would be the maximum permissible number of inference steps, as indicated in Supplementary Fig. 8a and Supplementary Table 2.

Alternatively, the maximum number of inference steps can be determined using information transfer limits. Typically, the optimized information transfer limit for aberration-corrected ADF-STEM images is around 60-70 pm for a STEM equipped with a fifth-order aberration corrector operating at 200 kV^6^. Based on this, we consider a 70 pm information transfer limit as a suitable criterion for determining the maximum number of inference steps.

**Reference**

1. Ho, J., Jain, A. & Abbeel, P. Denoising diffusion probabilistic models. In: *Proceedings of the 34th International Conference on Neural Information Processing Systems*). Curran Associates Inc. (2020).

2. Lu, C. *et al.* Diffusion-based deep learning method for augmenting ultrastructural imaging and volume electron microscopy. *Nat Commun* **15**, 4677 (2024).

3. Lyu, X. & Ren, X. Microstructure reconstruction of 2D/3D random materials via diffusion-based deep generative models. *Sci Rep* **14**, 5041 (2024).

4. Chung, H., Kim, J., Mccann, M. T., Klasky, Marc Louis, & Ye, J. C. Diffusion Posterior Sampling for General Noisy Inverse Problems. In: *International Conference on Learning Representations*) (OpenReview.net, 2023).

5. Hu, E. J. *et al.* LoRA: Low-Rank Adaptation of Large Language Models. Preprint at <https://arxiv.org/abs/2106.09685> (2021).

6. Isabell, T. *et al.* Development of a 200kV Atomic Resolution Analytical Electron Microscope. *Microscopy Today* **17**, 8-11 (2009).
